# Mathematical modelling of cytokines, MMPs and fibronectin fragments in osteoarthritic cartilage

**DOI:** 10.1007/s00285-017-1104-y

**Published:** 2017-02-17

**Authors:** Michelle Baker, Bindi S. Brook, Markus R. Owen

**Affiliations:** 1 School of Computer Science, University of Nottingham, Nottingham, UK; 2 Centre for Mathematical Medicine and Biology, School of Mathematical Sciences, University of Nottingham, Nottingham, UK

**Keywords:** Osteoarthritis, Cytokine, Modelling, Simulation, Non-linear dynamics, 92B05

## Abstract

Osteoarthritis (OA) is a degenerative disease which causes pain and stiffness in joints. OA progresses through excessive degradation of joint cartilage, eventually leading to significant joint degeneration and loss of function. Cytokines, a group of cell signalling proteins, present in raised concentrations in OA joints, can be classified into pro-inflammatory and anti-inflammatory groups. They mediate cartilage degradation through several mechanisms, primarily the up-regulation of matrix metalloproteinases (MMPs), a group of collagen-degrading enzymes. In this paper we show that the interactions of cytokines within cartilage have a crucial role to play in OA progression and treatment. We develop a four-variable ordinary differential equation model for the interactions between pro- and anti-inflammatory cytokines, MMPs and fibronectin fragments (Fn-fs), a by-product of cartilage degradation and up-regulator of cytokines. We show that the model has four classes of dynamic behaviour: homoeostasis, bistable inflammation, tristable inflammation and persistent inflammation. We show that positive and negative feedbacks controlling cytokine production rates can determine either a pre-disposition to OA or initiation of OA. Further, we show that manipulation of cytokine, MMP and Fn-fs levels can be used to treat OA, but we suggest that multiple treatment targets may be essential to halt or slow disease progression.

## Introduction

Osteoarthritis (OA) is a degenerative disease of the joints and is a leading cause of disability worldwide. Characterised by pain and stiffness in joints, OA leads to a significant loss of mobility. OA is strongly correlated with increasing age, with estimates that OA affects as many as 80% of those over 75 years of age (Arden and Cooper 2005). Other major risk factors include genetic predisposition, obesity, and injury or trauma to the joint (Felson et al. 2000). Recent research has also implicated inflammatory processes in the pathogenesis and progression of OA (Hedbom and Huselmann 2002; Scanzello and Goldring 2012).

The main disease mechanism in OA is the degeneration of the cartilage lining the joint. This allows the bone ends to rub together and eventually the whole joint is compromised and breaks down. Onset of OA varies between individuals and can be fast, known as acute onset OA. Typically, however, the onset will be gradual, with sporadic periods of symptoms followed by asymptomatic periods for several years as the condition progresses.

Despite a desperate need for disease-modifying treatments for OA, currently none have progressed beyond clinical trials (Roubille et al. 2015), and only pain-relieving treatments are available. There is a need for research into effective treatment in early OA to slow the progression of the disease.

Initiation of OA is the loss of the homoeostatic balance between extracellular matrix (ECM) synthesis and degradation (Sandell and Aigner 2001). Cartilage breakdown is accelerated through raised pro-inflammatory cytokine levels, stimulating production of matrix metalloproteinases (MMPs), which degrade the tissue (Martel-Pelletier et al. 2008). This degradation includes fibronectin breakdown and waste fibronectin fragments (Fn-fs) act as an irritant stimulating further pro-inflammatory cytokine response (Martel-Pelletier 2004). Pro-inflammatory cytokines and Fn-fs also induce production of anti-inflammatory cytokines offsetting some of this activity.

To date there are few published mathematical models of cytokine interactions specifically in OA but models which consider cartilage lesions, cytokine networks and inflammation may be relevant. A model of articular cartilage lesion formation and recovery (Wang et al. 2015; Graham et al. 2012), showed that in healthy tissue the balance of pro- and anti-inflammatory signalling is the determining factor in returning the system to homoeostasis after a blunt force injury. The model we present here also considers this balance but focuses on behaviour when the balance is lost. A model of acute systemic inflammation as a result of pathogen infection was presented by Kumar et al. (2004). The model identified five possible outcomes of infection some of which seem similar to those present in OA, particularly healthy response and recurrent inflammation. A general model of inflammation was proposed by Herald (2010) considering macrophage-mediated inflammation. The model showed that even small cytokine-dependent inflammatory responses to infection may become chronic rather than being resolved.

Two key models of cytokine signalling may be relevant to OA. One is a model of the dynamics of IL-1, TNF-$\alpha$ and IL-10 in monocytes (Seymour and Henderson 2001). The model showed different types of behaviour dependent upon the parameter values, including uncontrolled production of IL-1, stable equilibria and stable limit cycles which the authors linked to observed behaviour in RA and Septic Shock. A related model by Jit et al. (2005) looked in more depth at pro-inflammatory TNF-$\alpha$ and modelled the effects of anti TNF-$\alpha$ drugs in the inflamed synovial joint. From the model results the authors suggested that cytokine levels in RA were usually in equilibrium and anti TNF-$\alpha$ forced a shift from a disease equilibrium to a healthy equilibrium. However, they suggested that SIRS was a non-equilibrium condition and as such could not be moved to a healthy equilibrium state. A model of epithelium homoeostasis by Domínguez-Hüttinger et al. (2013) shows the importance of positive and negative feedback in homoeostatic regulation of inflammation.

We have previously published a two-variable ODE model analysing the interactions of cytokines (Baker et al. 2013) classified into two groups, pro- and anti-inflammatory. We showed that the feedback mechanisms gave rise to monostable and bistable behaviour, which we were able to associate with disease processes within the RA synovium. We suggested possible mechanisms of disease progression in RA as well as implications for treatment strategies. In that previous model we considered cytokine dynamics in isolation. However, we know that in OA other factors such as mechanical damage can lead to OA initiation and progression. Given that anti-cytokine therapies have so far proved unsuccessful in OA we suggest that not only the cytokine network but its relationship to other factors in OA, such as the waste products of joint damage, may be crucial in perpetuating the feedback networks leading to disease. For this reason, we extend this previous model to include MMPs and Fn-fs and investigate the interactions between these variables.

In the following section, we describe the model development, non-dimensionalisation and analysis of the steady states of the system. We also look at parameter sensitivity. In Sect. 3 we conduct a bifurcation analysis of the model, which shows the range of behaviours in the model which we classify based on the nature and stability of the steady states. In Sect. 4, we look at possible treatment strategies for the categories of behaviour observed in the bifurcation analysis. Finally, in Sect. 5, we discuss the main findings of the model and their implications.

## Model equations and steady state analysis

**Fig. 1:**
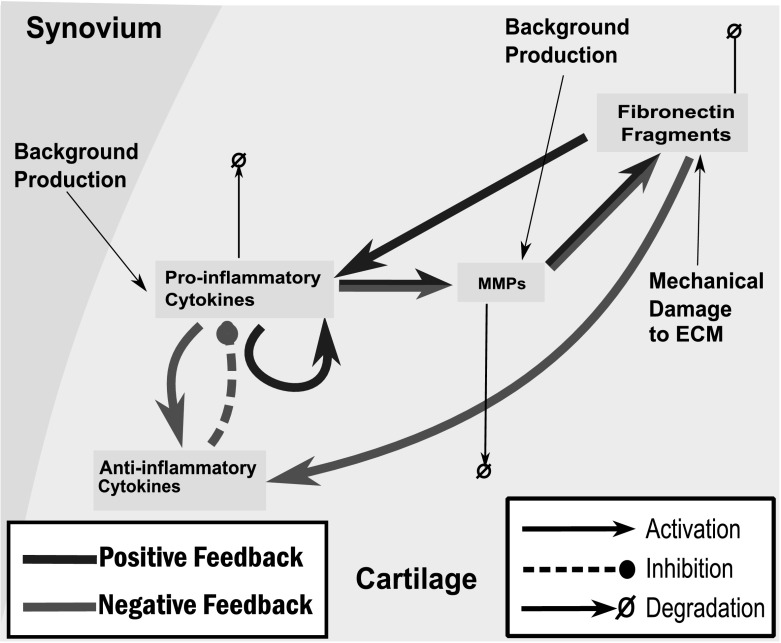
A simplified network of cytokine interactions within articular cartilage. Cytokines are classed as either pro- or anti-inflammatory. MMPs directly degrade the ECM leading to the release of fibronectin fragments (Fn-fs). Fn-fs are an irritant leading to an increased cytokine response

Our four model variables are pro-inflammatory cytokines (*p*), anti-inflammatory cytokines (*a*), MMPs (*m*) and Fn-fs (*f*). The network of interactions between these variables is shown in Fig. 1 and is based on the joint and cytokine biology as described in Wojdasiewicz et al. (2014), Fernandes et al. (2002), Goldring (2000b) and Westacott and Sharif (1996).

Pro-inflammatory cytokines are normally present at low levels in the cartilage as they play a role in mediating the normal turnover of the ECM (Poole 1993), which involves remodelling the cartilage ECM at a very slow rate to maintain tissue integrity. Production of pro-inflammatory cytokine is up-regulated in response to trauma or infection, as part of the inflammatory response and repair mechanism. This response is usually kept in homoeostatic balance by anti-inflammatory cytokines, which act both to inhibit the synthesis of pro-inflammatory cytokines and also to block pro-inflammatory cytokine receptors (Opal and DePalo 2000). To model these processes we assume that production of pro-inflammatory cytokines, *p*, is dependent on itself, anti-inflammatory cytokines, *a*, and Fn-fs, *f*,

1 $$\begin{aligned} \frac{\mathrm{d} p}{\mathrm{d} t}&= \left( c_0 + c_1 \frac{p^n}{{c_2}^n + p^n} + c_3 \frac{f^n}{{c_4}^n+f^n}\right) \left( \frac{{c_5}^n}{{c_5}^n + a^n} \right) - d_p p. \end{aligned}$$

We assume that production rates will be limited biologically and therefore model functions of *p*, *a* and *f* as saturating Hill functions with Hill coefficients, *n*. For simplicity in Eq. 1 and the following we assume equal Hill coefficients in all regulatory Hill functions. In the discussion we consider the implications of unequal Hill coefficients. We also assume that pro-inflammatory cytokines will degrade naturally at rate $d_p$. We choose to make the stimulatory terms additive since these two pathways are biochemically distinct and activate different cell receptors. This means that even if there is no cartilage degradation there may still be a large cytokine response due to an increase in *p*. Since anti-inflammatory cytokines reduce production and effectiveness of pro-inflammatory cytokines regardless of the source we apply the anti-inflammatory inhibition term to all the source terms.

Anti-inflammatory cytokine production is stimulated by both pro-inflammatory cytokines and fibronectin fragments. The dynamics of anti-inflammatory cytokines, *a*, therefore includes source terms (saturating Hill functions of *p* and *f*) representing their up-regulation by both pro-inflammatory cytokines and Fn-fs, and a natural degradation term,

2 $$\begin{aligned} \frac{\mathrm{d} a}{\mathrm{d} t}&= c_6 \frac{p^n}{{c_7}^n+p^n} + c_8 \frac{f^n}{{c_{9}}^n+f^n} - d_a a. \end{aligned}$$

MMPs mediate ECM degradation and the synthesis of MMPs is stimulated by pro-inflammatory cytokines (Vincenti and Brinckerhoff 2002). MMPs are also found at low levels in normal cartilage so we assume some basal production. Together with natural degradation, the dynamics of MMPs (*m*) are therefore modelled by

3 $$\begin{aligned} \frac{\mathrm{d} m}{\mathrm{d} t}&= c_{10} + c_{11} \frac{p^n}{{c_{12}}^n + p^n} - d_m m. \end{aligned}$$

We model Fn-fs production as a result of ECM degradation at a rate proportional to the MMP level, *m*, with an additional constant rate $c_{14}$ representing mechanical damage. We also include natural degradation, giving

4 $$\begin{aligned} \frac{\mathrm{d} f}{\mathrm{d} t}&= c_{13} m + c_{14} - d_f f. \end{aligned}$$

We non-dimensionalise the model using the scalings:

$$\begin{aligned} p = c_{2}{\tilde{p}} \quad a = c_5 {\tilde{a}} \quad m = \frac{{\tilde{m}} c_4 d_a}{c_{13}} \quad f = c_4 {\tilde{f}} \quad t = \frac{{\tilde{t}}}{d_a} \end{aligned}$$

where the tilde denotes dimensionless quantities. Time is scaled with the degradation of anti-inflammatory cytokines, which we expect to be in the order of minutes. Dropping the tildes for convenience gives the dimensionless model:

5 $$\begin{aligned} \frac{\mathrm{d} p}{\mathrm{d} t}&= \left( P_{bp}+ P_{pp}\frac{p^n}{1 + p^n} + P_{fp}\frac{f^n}{1+f^n}\right) \left( \frac{1}{1 + a^n} \right) - \gamma _p p \end{aligned}$$

6 $$\begin{aligned} \frac{\mathrm{d} a}{\mathrm{d} t}&= A_{pp}\frac{p^n}{{A_{ph}}^n+p^n} + A_{fp}\frac{f^n}{A_{fh}^n+f^n} - a \end{aligned}$$

7 $$\begin{aligned} \frac{\mathrm{d} m}{\mathrm{d} t}&= M_{bp}+ M_{pp}\frac{p^n}{{M_{ph}}^n + p^n} - \gamma _m m \end{aligned}$$

8 $$\begin{aligned} \frac{\mathrm{d} f}{\mathrm{d} t}&= m + F_{dam}- \gamma _f f \end{aligned}$$

where,

$$\begin{aligned} P_{bp}&= \frac{c_0}{c_{2} d_a}, \qquad P_{pp}= \frac{c_1}{c_{2} d_a}, \qquad P_{fp}= \frac{c_3}{c_2 d_a}, \qquad A_{pp}= \frac{c_6}{c_{5} d_a},\\ A_{ph}&= \frac{c_7}{c_2}, \qquad A_{fp}= \frac{c_8}{c_5d_a}, \qquad A_{fh}= \frac{c_{9}}{c_4}, \qquad M_{bp}= \frac{c_{10} c_{11}}{c_4 {d_a}^2}, \\ M_{pp}&= \frac{c_{11} c_{13}}{c_4 {d_a}^2}, \qquad M_{ph}= \frac{c_{12}}{c_{2}}, \qquad F_{dam}= \frac{c_{14}}{c_4 d_a}, \qquad \gamma _p = \frac{d_p}{d_a},\\ \gamma _m&= \frac{d_m}{d_a}, \qquad \gamma _f = \frac{d_f}{d_a}. \end{aligned}$$

The meaning of each of these new parameters is summarised in Table 1.

**Table 1:** The parameters in the system (5)–(8), their interpretation and the reference values used throughout the paper

Parameter	Description	Value
$P_{bp}$	Background pro-inflammatory production	0.01
$P_{pp}$	Pro-inflammatory cytokine driven pro-inflammatory cytokine production	10
$P_{fp}$	Fibronectin fragment driven pro-inflammatory cytokine production	10
$A_{pp}$	Pro-inflammatory cytokine driven anti-inflammatory cytokine production	10
$A_{ph}$	Pro-inflammatory cytokine concentration at which pro-inflammatory cytokine driven anti-inflammatory cytokine production is half maximal	1
$A_{fp}$	Fibronectin fragment driven anti-inflammatory cytokine production	10
$A_{fh}$	Concentration at which Fibronectin fragment driven anti-inflammatory cytokine production is half maximal	1
$M_{bp}$	Background MMP production	0.01
$M_{pp}$	Pro-inflammatory cytokine driven MMP production	10
$M_{ph}$	Pro-inflammatory cytokine concentration at which MMP production is half maximal	1
$F_{dam}$	Mechanical damage parameter	0
$\gamma _p$	Relative rate of clearance of pro-inflammatory cytokine to anti-inflammatory cytokine	1
$\gamma _m$	Relative rate of clearance of MMP to anti-inflammatory cytokine	1
$\gamma _f$	Relative rate of clearance of fibronectin fragments to anti-inflammatory cytokine	1
*n*	Hill coefficients of regulatory Hill functions	2

Insight into the nature of the steady states of this system can be gained from the nullclines, which are hypersurfaces given by

9 $$\begin{aligned} \dot{p} = 0 \Longleftrightarrow a&= \left( \frac{1}{\gamma _p p}\left( {P_{bp}+ P_{pp}\frac{p^n}{1+p^n} + P_{fp}\frac{f^n}{1+f^n}}\right) -1\right) ^{\frac{1}{n}}, \end{aligned}$$

10 $$\begin{aligned} \dot{a} = 0 \Longleftrightarrow a&=A_{pp}\frac{p^n}{A_{ph}^n+p^n} + A_{fp}\frac{f^n}{A_{fh}^n+f^n},\end{aligned}$$

11 $$\begin{aligned} \dot{m} = 0 \Longleftrightarrow m&= \frac{M_{bp}}{\gamma _m} + \frac{M_{pp}}{\gamma _m} \frac{p^n}{M_{ph}^n + p^n},\end{aligned}$$

12 $$\begin{aligned} \dot{f} = 0 \Longleftrightarrow f&= \frac{m + F_{dam}}{\gamma _f}. \end{aligned}$$

The steady states of the model are the points where all the nullclines intersect. Substituting Eqs. 11 and 12, $m=N_m(p)$ and $f=N_f(N_m(p))$ into 9 and 10 reduces the problem to two simultaneous equations for *a* in terms of *p*:

13 $$\begin{aligned} a = N_p(p) = \left( \frac{h(p)}{\gamma _p p}-1 \right) ^{\frac{1}{n}}, \end{aligned}$$

where

14 $$\begin{aligned} h(p)&=P_{bp}+ P_{pp}\frac{p^n}{1+p^n} \nonumber \\&\quad + P_{fp}\frac{\left( F_{dam}\gamma _m + M_{bp}+ M_{pp}\frac{p^n}{M_{ph}^n + p^n} \right) ^n}{({\gamma _f \gamma _m})^n+\left( F_{dam}\gamma _m + M_{bp}+ M_{pp}\frac{p^n}{M_{ph}^n + p^n} \right) ^n}, \end{aligned}$$

and

15 $$\begin{aligned} a&= N_a(p) = A_{pp}\frac{p^n}{A_{ph}^n+p^n} \nonumber \\&\quad + A_{fp}\frac{\left( F_{dam}\gamma _m + M_{bp}+ M_{pp}\frac{p^n}{M_{ph}^n + p^n} \right) ^n}{(\gamma _f \gamma _m)^nA_{fh}^n +\left( F_{dam}\gamma _m + M_{bp}+ M_{pp}\frac{p^n}{M_{ph}^n + p^n} \right) ^n}. \end{aligned}$$

The intersections of $N_p(p)$ and $N_a(p)$, given by Eqs. (13) and (15), give the steady states, although analytical solutions are in general not tractable. The forms of Eqs. (13) and (15) mean that they will always intersect at least once, so the system will always have at least one steady state. With a Hill coefficient of $n = 1$, Eq. (15) increases monotonically and Eq. (13) decreases monotonically, giving only one possible steady state. For $n > 2$, the nullclines take a similar form as with n $=$ 2, although the parameter values differ and the steeper gradients allow for the possibility of additional steady states, in smaller regions of parameter space. For this reason we focus on the case $n = 2$ as in our previous work, Baker et al. (2013), through the rest of this analysis.

**Fig. 2:**
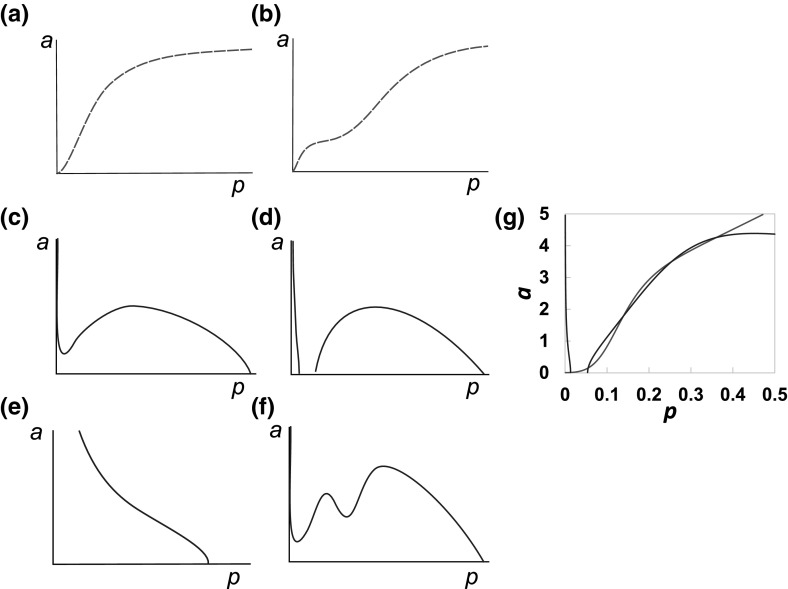
Possible forms of the $N_p(p)$ and $N_a(p)$, whose intersections define steady states of the OA-model [Eqs. (5)–(8)]. **a**, **b** Schematic representation showing how $N_a(p)$ can be sigmoidal (**a**) or double-sigmoidal (**b**). **c**–**f** $N_p(p)$ can take several forms that may meet the *p*-axis one (**c**, **e**, **f**) or three (**d**) times. **g** A specific example showing both $N_p(p)$ and $N_a(p)$ which cross five times, using the parameters $P_{bp}=M_{bp}=0.01, P_{pp}=P_{fp}=A_{pp}=M_{pp}=10, A_{fp}=3.2, A_{ph}=M_{ph}=1, A_{fh}=0.2, \gamma _p=\gamma _f=\gamma _m=1$ and $F_{dam}=0$

The first term in Eq. (15) is a Hill function of *p* and the second is a Hill function of *p* embedded within another Hill function. This allows $N_a(p)$ to take two qualitative forms, either a sigmoidal shape, if $A_{ph}$ and $A_{fh}$ are close in value (Fig. 2a), or a double sigmoidal shape if $A_{ph}$ and $A_{fh}$ are sufficiently different (Fig. 2b).

Equation (13) is only valid when $h(p)\ge \gamma _p p$. Since $h(0)>0$ (for positive basal production), as $\lim _{p\rightarrow \infty } \frac{h(p)}{\gamma _pp}$ and *h*(*p*) is continuous, $N_p(p)$ always meets the *p*-axis for a large enough value of *p*.

The function *h*(*p*), similar to Eq. (15), can have either a sigmoidal or double sigmoidal shape. The double sigmoidal shape is possible when the terms involving $P_{pp}$ and $P_{fp}$ are sufficiently different. This leads to several possible shapes of $a = N_p(p) = \sqrt{\frac{h(p)}{\gamma _p p}-1}$. These are illustrated qualitatively in Fig. 2(c–f).

It is clearly possible for these curves to also intersect three times. We have found a maximum of five (see Sect. 3.5) with the Hill coefficients of $n = 2$ but could conceivably have more. Two or four steady states will only occur when the curves meet tangentially. Since this only occurs at bifurcations we will focus on cases with one, three or five steady states.

**Fig. 3:**
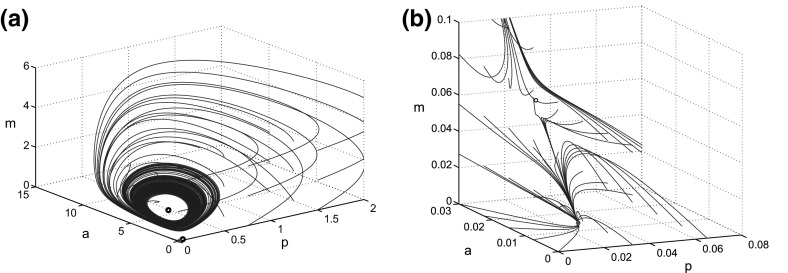
For the reference parameter set (Table 1), the OA model has a stable quiescent state and a stable inflamed limit cycle. The figure shows a projection of the phase space for the reference parameter set (Table 1), showing trajectories for the cartilage model [Eqs. (5)–(8)] for various regularly spaced initial conditions in (*p*, *a*, *m*) space. **a** Shows all three steady states whilst, **b** focuses on the behaviour around the quiescent steady state. The *black circles* show the position of unstable fixed points and the *red dot* shows the stable fixed point. The trajectories either move to the stable fixed point or the stable limit cycle which surrounds an unstable fixed point. The unstable steady state influences the path taken by trajectories (colour figure online)

Due to the difficulties of obtaining measurements of cytokine levels and rates both in vivo and in vitro, there is little data concerning the parameters in the model. We therefore chose an illustrative initial parameter set, summarised in Table 1, in which similar processes have similar rates and concentration dependence, in the absence of data suggesting otherwise. The relative thresholds in Hill functions ($A_{ph}, A_{fh}, M_{ph}$) are all set to one, equal to the dimensionless thresholds for pro-inflammatory cytokine regulation in Eq. 5. Similarly, the relative degradation rates ($\gamma _p, \gamma _m, \gamma _f$) are equal to the dimensionless degradation of *a*. We set all the cytokine and MMP production parameters ($P_{pp}, P_{fp},A_{pp},A_{fp}, M_{pp}$) to 10 and background rates ($P_{bp}, M_{bp}$) to 0.01 in order that regulated production is relevant and not dominated by background rates. These choices avoid unnecessary bias in the network. In the following sections we consider parameter variations about this initial set in order to uncover the relevant biologically plausible behaviours to be expected from the system. Variations reasonably close to this reference parameter set show the range of behaviours that the model can display. These can be classified into four robust behaviour groups: homoeostasis, bistable inflammation, tristable inflammation and persistent inflammation. These groups may better align to individuals clinical manifestation of the disease and we propose that the behavioural group would be the determinant for long term prognosis.

With these parameters the system is bistable with three steady states: $S_0, S_1$ and $S_2$. When the levels of *p* are low, as at $S_0$,which is stable, we assume this would indicate a quiescent steady state, where cells are producing only the basal level of *p* necessary for homoeostatic matrix turnover. The stable limit cycle around the unstable state $S_2$ is likely to indicate an inflamed state due to high and fluctuating levels of *p* and *f*. A projection of the phase-space for this system is shown in Fig. 3a, b, with the latter showing the steady states in more detail. The diagrams suggest that the stable inflamed limit cycle has a large basin of attraction whilst the quiescent steady state has only a small basin of attraction.

**Fig. 4:**
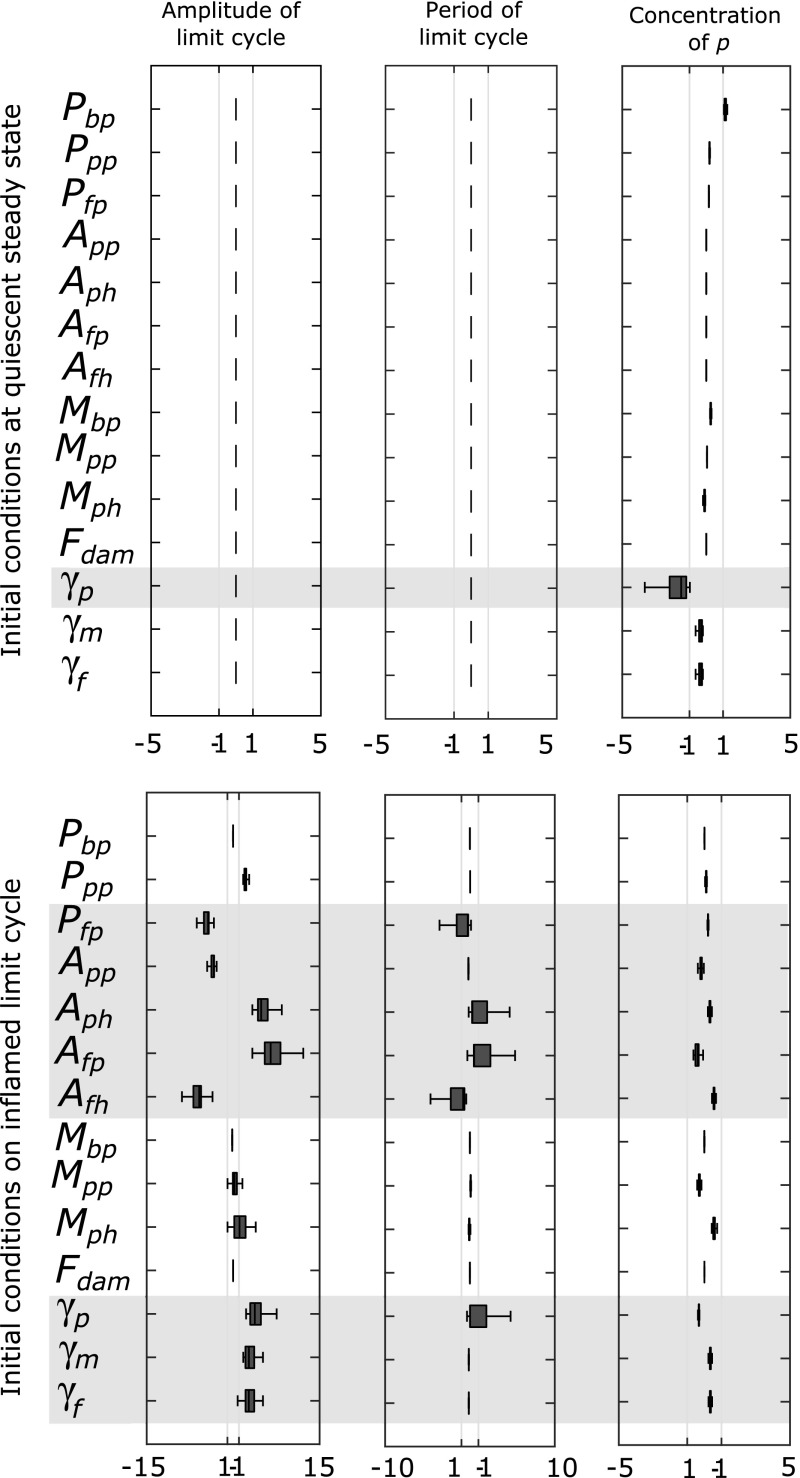
Parameters related to anti-inflammatory cytokine production are particularly sensitive. Box-plots of the sensitivity of steady state pro-inflammatory cytokine (*p*) and the period and amplitude of limit cycles, to variations in model parameters. The *top row* shows sensitivity at a quiescent state whilst the *bottom row* shows sensitivity at an inflamed state. *Boxes* show the interquartile range (IQR) of the relative sensitivity coefficient as the parameters are varied randomly from a uniform distribution within the range [−30%, 30%] (n $=$ 1000). A value of 1 is representative of an equal change in the feature for a given change in the parameter

A sensitivity analysis of small parameter changes around the reference parameter set gives some insight into the behaviour of the system. Figure 4 shows sensitivity of the parameters to changes of up to $\pm 30\%$ as measured by three features: the concentration of *p* at the steady state, the amplitude and the period of any limit cycles. We use a one-at-a-time sensitivity analysis to measure the sensitivity gain for each feature according to the sensitivity function,

16 $$\begin{aligned} S^\phi _k = \frac{\delta \phi / \phi }{\delta k / k}, \end{aligned}$$

where $\phi$ is the feature being measured and *k* is the parameter being changed. We repeat this for 1000 iterations choosing a uniformly distributed random percentage change each time within the range [−30%, 30%]. Parameters related to anti-inflammatory cytokine production ($A_{pp},A_{ph},A_{fp},A_{fh}$) and clearance rate parameters ($\gamma _p,\gamma _m,\gamma _f$) are consistently the most sensitive when the system is at the inflamed state. This suggests that if we alter these parameters from the reference parameter set we may have significantly different behaviour. For the parameters $P_{fp}, A_{pp}$ and $A_{fh}$ the sensitivity coefficient is negative indicating that increases in these parameter values lead to a smaller limit cycle amplitude. Whereas, for the other sensitive parameters the coefficient is positive hence an increase in these parameters leads to a larger limit cycle amplitude. At the quiescent state the system is generally robust to parameter changes up to ±30% with the exception of changes to $\gamma _p$ which can move the system from the quiescent to the inflamed state with parameter changes within this range. We will consider this in Sect. 3.6.

## Bifurcation analysis

**Fig. 5:**
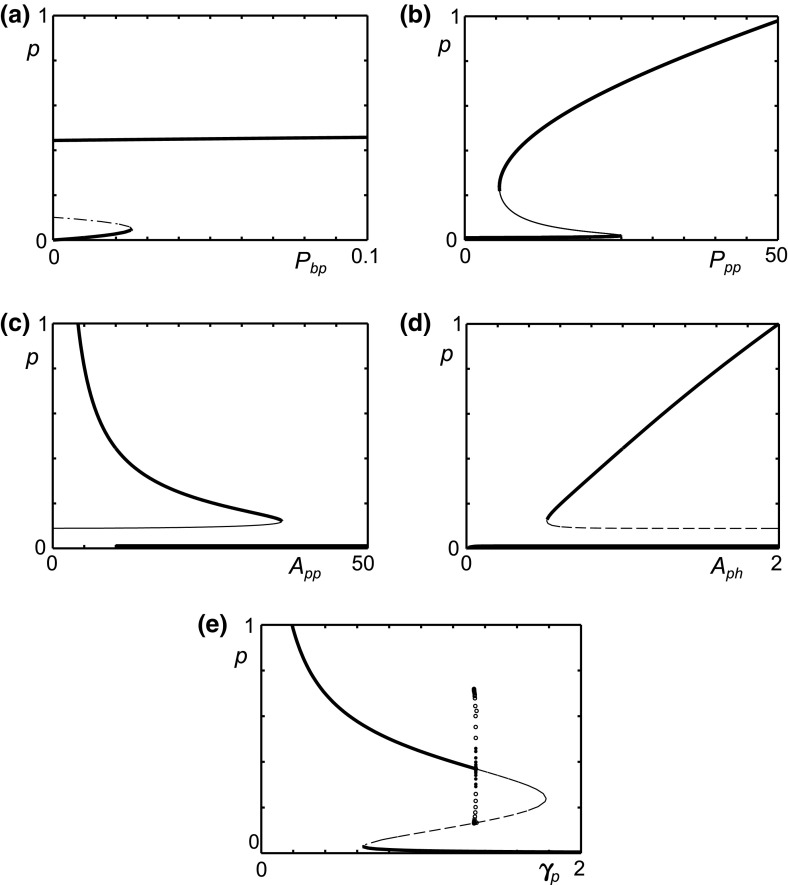
Bifurcation diagrams of the cytokine only model differentiate cytokine-driven from fibronectin-driven effects. Bifurcation diagrams of the cytokine-only model, (Baker et al. 2013), using the reference parameter values used in this paper showing the transitions from monostable to bistable. This model does not have any fibronectin involvement so comparison of **a**, **b** with Figs. 6a, b and **c**, **d**, **e** with 10a, c, e respectively, shows the effect of the fibronectin fragment driven feedback

In this section we examine the behaviour of the steady states as we vary the parameters. In the limit when $M_{bp}=0$ and $M_{pp}=0$, this model reduces to the cytokine-only model of Baker et al. (2013). Figure 5 shows bifurcation diagrams for the parameters which are present in both models, with $M_{bp}=0$ and $M_{pp}=0$. Here we can see how changes to the cytokine-related parameters can result in transitions between homoeostasis and inflammation without any mechanical component. Throughout this section we will compare the current model with the cytokine-only model to see the effect of fibronectin-mediated feedback.

Since there are fourteen parameters in this model, we focus on those parameters that we believe may have a bearing on OA initiation and progression. Pro-inflammatory cytokine production ($P_{bp}, P_{pp}, P_{fp}$), MMP production ($M_{bp}, M_{pp}$), Fn-fs production ($F_{dam}$) and Fn-fs clearance ($\gamma _f$) have all been implicated in disease progression and are considered potential treatment targets (Pearle et al. 2005; Homandberg et al. 1998; Martel-Pelletier et al. 2008). The remaining parameters, those relating to anti-inflammatory production, cytokine and MMP clearance and half maximal parameters, are discussed more briefly, with figures in the “Appendix”, since we are not considering these as possible treatment targets.

Henceforth, we use the level of *p* as a measure of whether an individual is in a quiescent or inflamed state. A quiescent state, with low *p*, would indicate that either (i) an individual is healthy and shows no signs of OA or (ii) an individual has OA but the disease is not actively progressing at that time (i.e. the individual is not in a period of *flare up*). An inflamed state, with high *p*, would indicate that cartilage is being actively degraded and there is at least sub-clinical inflammation, but would not necessarily indicate clinically observable levels of inflammation. In this model we see four classifications of behaviour: homoeostasis, where we have only a quiescent state and any trajectories perturbed away from this state will return; persistent inflammation where we have only an inflamed state; bistable inflammation and tristable inflammation. In the multi-stable cases, we have both a quiescent and either one or two inflamed states, and, given appropriate perturbation in the model, transitions between the states can occur. A summary of behaviour types found in the model can be found Table 2.

**Table 2:** Summary of the behaviours that arise for different values of the parameters in system (5)–(8)

Name	No. steady states	Stability of steady states	Limit cycles	Type
Ai	1	$S_0^S$	–	Homoeostasis
Aii	1	$S_0^S$	–	Persistent inflammation
Aiii	1	$S_0^U$	$L_1^S$	Persistent inflammation
Bi	3	$S_0^S, S_1^U, S_2^U$	–	Homoeostasis
Bii	3	$S_0^U, S_1^U, S_2^S$	–	Persistent inflammation
Biii	3	$S_0^S, S_1^U, S_2^S$	–	Bistable
Ci	3	$S_0^S, S_1^U, S_2^U$	$L_1^S$	Bistable
Cii	3	$S_0^U, S_1^U, S_2^S$	$L_1^S$	Bistable
Ciii	3	$S_0^S, S_1^U, S_2^S$	$L_1^S$	Bistable
Civ	3	$S_0^S, S_1^U, S_2^S$	$L_1^U, L_2^S$	Bistable
Cv	3	$S_0^U, S_1^U, S_2^U$	$L_1^S$	Persistent inflammation
Di	3	$S_0^S, S_1^U, S_2^S$	$L_1^U, L_2^S$	Bistable
Dii	3	$S_0^S, S_1^U, S_2^U$	$L_1^S, L_2^U$	Bistable
Diii	3	$S_0^U, S_1^U, S_2^S$	$L_1^U, L_2^S$	Bistable
Ei	5	$S_0^S, S_1^U, S_2^S, S_3^U, S_4^S$	–	Tristable
Eii	5	$S_0^S, S_1^U, S_2^U, S_3^U, S_4^S$	–	Bistable
Fi	5	$S_0^S, S_1^U, S_2^U, S_3^U, S_4^S$	$L_1^S$	Tristable
Fii	5	$S_0^S, S_1^U, S_2^S, S_3^U, S_4^S$	$L_1^U$	Tristable

The abbreviation S means Stable and U means Unstable, indicating the stability of the steady state or limit cycle

### Loss of the quiescent steady state

Since the quiescent steady state of the system occurs only when all the variables are small we can simplify the system [Eqs. (5)–(8)] to analyse the loss of this steady state. To find the leading order terms of Eq. (5) around the reference parameter set we rescale all the parameters using the small parameter $\epsilon$ where $\epsilon \sim 0.01$ such that all the hatted parameters are O(1). If *a* is small we lose the anti-inflammatory inhibition term since it is approximately 1. Additionally, if *f* is small Eq. (8) is a quadratic in *p*, hence Eq. (5) in the limit when all the variables are small, becomes,

17 $$\begin{aligned} \epsilon \frac{\mathrm{d} {\widehat{p}}}{\mathrm{d} t} =&\epsilon {\widehat{P_{bp}}} + \frac{\epsilon ^2}{\sqrt{\epsilon }}{\widehat{P_{pp}}} {\widehat{p}}^2 + \frac{\epsilon ^3}{\sqrt{\epsilon }}\frac{{\widehat{P_{fp}}}{\widehat{M_{pp}}}^2}{\widehat{\gamma _m}^2\widehat{\gamma _f}^2{\widehat{M_{ph}}}^4}{\widehat{p}}^4 +2\epsilon ^2\frac{{\widehat{P_{fp}}}{\widehat{M_{bp}}}{\widehat{M_{pp}}}}{\widehat{\gamma _m}^2\widehat{\gamma _f}^2{\widehat{M_{ph}}}^2} {\widehat{p}}^2 \nonumber \\&+ 2\epsilon ^2\frac{{\widehat{P_{fp}}}{\widehat{M_{pp}}}{\widehat{F_{dam}}}}{\widehat{\gamma _m}\widehat{\gamma _f}^2{\widehat{M_{ph}}}^2} {\widehat{p}}^2 -\epsilon \widehat{\gamma _p} {\widehat{p}} +\frac{\epsilon ^2}{\sqrt{\epsilon }}\frac{{\widehat{M_{bp}}}^2{\widehat{P_{fp}}}}{\widehat{\gamma _m}^2\widehat{\gamma _f}^2} \nonumber \\&+\frac{2\epsilon ^2}{\sqrt{\epsilon }}\frac{{\widehat{P_{fp}}}{\widehat{M_{bp}}}{\widehat{F_{dam}}}}{\widehat{\gamma _m}\widehat{\gamma _f}^2}+\frac{\epsilon ^2}{\sqrt{\epsilon }}\frac{{\widehat{P_{fp}}}{\widehat{F_{dam}}}^2}{\widehat{\gamma _f}^2}=0, \end{aligned}$$

where,

$$\begin{aligned} p&= \epsilon {\widehat{p}},&f&=\epsilon {\widehat{h}},&P_{bp}&= \epsilon {\widehat{P_{bp}}},\\ P_{pp}&= \frac{{\widehat{P_{pp}}}}{\sqrt{\epsilon }},&P_{fp}&= \frac{{\widehat{P_{fp}}}}{\sqrt{\epsilon }},&M_{bp}&= \epsilon {\widehat{M_{bp}}},\\ M_{pp}&= \frac{{\widehat{M_{pp}}}}{\sqrt{\epsilon }},&M_{ph}&= {\widehat{Mph}},&F_{dam}&= \epsilon {\widehat{F_{dam}}},\\ \gamma _p&= \widehat{\gamma _p},&\gamma _m&= \widehat{\gamma _m},&\gamma _f&= \widehat{\gamma _f}=0. \end{aligned}$$

Taking leading order terms (O($\epsilon$)) and next order terms (O($\epsilon ^2 / \sqrt{\epsilon }$)) we have,

18 $$\begin{aligned} \frac{\mathrm{d} p}{\mathrm{d} t}&= {\widehat{P_{pp}}} {\widehat{p}}^2 -\widehat{\gamma _p} {\widehat{p}} + {\widehat{P_{bp}}} + \frac{{\widehat{M_{bp}}}^2{\widehat{P_{fp}}}}{\widehat{\gamma _m}^2 \widehat{\gamma _f}^2}+\frac{2{\widehat{P_{fp}}}{\widehat{M_{bp}}}{\widehat{F_{dam}}}}{\widehat{\gamma _m}\widehat{\gamma _f}^2}\nonumber \\&\quad + \frac{{\widehat{P_{fp}}} \widehat{F^2_{dam}}}{\widehat{\gamma _f}^2}=0 \end{aligned}$$

The discriminant of (18) determines where we switch from two roots to zero roots, which represents the loss of the small amplitude steady state. Hence we will lose the quiescent state if

19 $$\begin{aligned} \widehat{\gamma _p}&< {\widehat{P_{pp}}}\left( {\widehat{P_{bp}}} + \frac{{\widehat{M_{bp}}}^2{\widehat{P_{fp}}}}{\widehat{\gamma _m}^2 \widehat{\gamma _f}^2}+\frac{2{\widehat{P_{fp}}}{\widehat{M_{bp}}}{ \widehat{F_{dam}}}}{\widehat{\gamma _m}\widehat{\gamma _f}^2} + \frac{{\widehat{P_{fp}}}\widehat{{F^2_{dam}}}}{\widehat{\gamma _f}^2}\right) . \end{aligned}$$

This shows that reducing clearance rates ($\gamma _m$ or $\gamma _f$) or increasing production rates ($P_{bp}, P_{fp}, M_{bp}$ or $M_{pp}$) is likely to lead to inflammation. Mechanical damage is a key driver in moving the system towards inflammation, since the parameter $F_{dam}$ appears in two terms. In the absence of mechanical damage, cytokine- and fragment-driven feedback are key to driving the system to inflammation. Referring back to the network diagram, Fig. 1, this indicates that where we do not have mechanical damage, the cytokine-only feedback loops will dominate system behaviour and drive the move from quiescence. However, if we have mechanical damage, the MMP- and fibronectin-driven feedback loops become much more important and can become disease drivers even where the cytokine feedback parameters are at basal levels.

Decreases in $\gamma _p$ the left-hand side of the inequality [Eq. (19)] push the system towards inflammation. However, the clearance rate of *p* is determined by the half-life of the pro-inflammatory cytokines, which is unlikely to vary either with time or between individuals, so is unlikely to be a driver of inflammation in most cases.

### Pro-inflammatory cytokine production parameters

**Fig. 6:**
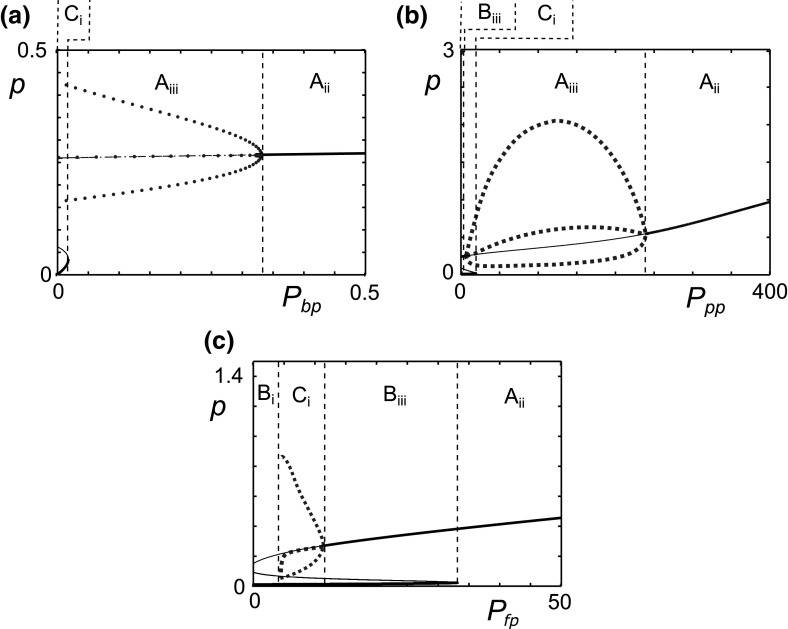
Increases in pro-inflammatory cytokine production lead to persistent inflammation. Bifurcation plots of the pro-inflammatory cytokine level (*p*) against pro-inflammatory cytokine production parameters **a** $P_{bp}$, **b** $P_{pp}$ and **c** $P_{fp}$. The *blue dotted lines* represent the minimum, maximum and average values of the limit cycles. The *vertical black dashed lines* denote the transition between different behaviours, which are labelled and summarised in Table 2 (colour figure online)

The parameters $P_{bp}, P_{pp}$ and $P_{fp}$ govern the production of pro-inflammatory cytokines. These cytokines are raised in OA and this has been implicated in disease progression (Hedbom and Huselmann 2002). The mechanism by which these raised levels occur is unclear but could be the result of higher than normal production rates of pro-inflammatory cytokines. If any of these three parameters are sufficiently high, bistability is lost via a fold bifurcation, and there is a single steady state (Fig. 6).

For intermediate $P_{bp}$ and $P_{pp}$ values, the loss of bistability is followed by a single stable limit cycle (Fig. 6a, b), representing an oscillatory inflamed state. Then at higher levels of these parameters this is lost, via a Hopf bifurcation, leaving a single stable steady state. For increases in $P_{fp}$, the Hopf bifurcation is encountered before the fold bifurcation, giving rise to two stable steady states for some values of $P_{fp}$. For low values of $P_{fp}$, we can have a single quiescent steady state representing homoeostasis. As the limit cycle collides with $S_1$ at a homoclinic bifurcation it leaves only one stable and two unstable steady states. Examination of the phase space suggests that, as for the reference parameter set, the basin of attraction of the inflamed state is large in the bistable region and remains large even as we move towards the homoclinic. Even in the homoeostasis region, trajectories undergo large fluctuations in *p* before settling to the quiescent state. One interpretation of these large basins of attraction is that major deviations from the state of quiescence due to trauma or infection are likely to move an individual to a state of inflammation, since the large basins for inflammation persist over the range of small parameter variations, which we might expect to see in different individuals. Even in homoeostasis large perturbations to the system take a long time to resolve, during which some tissue damage may accumulate. This behaviour may point to the reason for OA being so prevalent since the system trajectories deviate from the quiescent steady state for wide ranges of parameters and initial conditions.

Comparison of Fig. 5a, b (cytokine-only dynamics) with Fig. 6a, b shows similar behaviour. One important difference, however, is that both $P_{bp}$ and $P_{pp}$ in Fig. 6 show oscillatory behaviour whereas the inflamed states are fixed points in the cytokine-only model. If a fixed inflamed state is clinically preferable to an oscillatory one, for example if this was less symptomatic for patients, the model suggests that inhibition of fragment-driven feedback may be a justifiable treatment aim.

### MMP production parameters

**Fig. 7:**
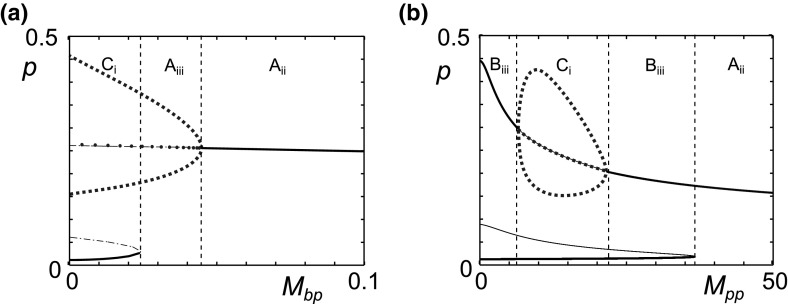
High rates of MMP production lead to persistent inflammation. Bifurcation diagrams of pro-inflammatory cytokine level (*p*) against the MMP production parameters $M_{bp}$ and $M_{pp}$. The *blue dotted lines* represent the minimum, maximum and average values of the limit cycles. The *vertical black dashed lines* denote the transition between different behaviours, which are labelled and summarised in Table 2

The parameters $M_{bp}$ and $M_{pp}$ determine the maximum rates of MMP production. Since MMP levels are known to be raised in OA these parameters are of great interest. Figure 7 shows that, for variations in these parameters about the reference parameter set, there are no regions of homoeostasis. At high levels of MMP production, with either high $M_{bp}$ or $M_{pp}$, there is a region of persistent inflammation with a stable steady state. In both cases, at lower production levels, including the reference parameter set, a fold bifurcation leads to bistability with the introduction of stable and unstable steady states, providing the possibility of moving to a quiescent state. For $M_{pp}$ (Fig. 7b), within the bistable region there is a region of oscillatory inflammation due to two Hopf points.

**Fig. 8:**
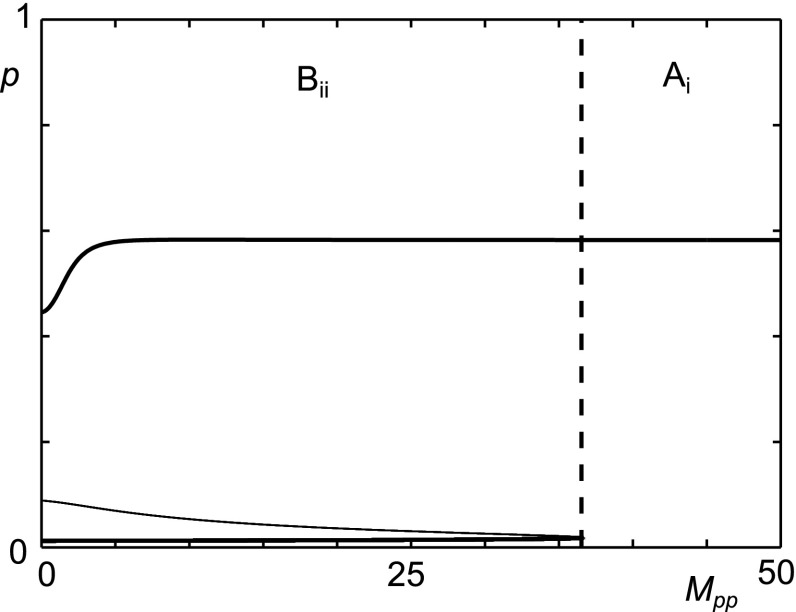
The MMP production rate, $M_{pp}$, is involved in both negative and positive feedback. Hence, its bifurcation behaviour is dependent on the parameter set. Reduced fibronectin driven anti-inflammatory cytokine production allows the positive feedback pathway to dominate, hence increases in $M_{pp}$ lead to higher values of *p* at the steady state. Bifurcation diagram of pro-inflammatory cytokine level (*p*) against the MMP production parameter $M_{pp}$. The value of $A_{fp}$ is reduced from 10 (seen in Fig. 7b) to 2 whilst all other parameters are the same as in the reference set. The *vertical black dashed lines* denote the transition between different behaviours, which are labelled and summarised in Table 2

For this parameter set, for an individual in an oscillatory inflamed state, an increase in MMP production pushes the system to a steady inflamed state in which *p* is lower than the average of the limit cycle in the oscillatory states. However, the level of *f* is lower at smaller values of $M_{pp}$ so the lower levels of *p* do not necessarily imply less cartilage degradation. This counter-intuitive result arises from the balance in the positive and negative feedback pathways. $M_{pp}$ is part of both pathways and for the reference parameter set the negative feedback is dominant. If we make the negative feedback weaker, by reducing the value of $A_{fp}$, the positive pathway dominates, and the value of *p* at the steady state increases with higher values of $M_{pp}$ as illustrated in Fig. 8.

### Fibronectin fragment related parameters

Fibronectin fragments have been shown to exacerbate cartilage degradation in OA (Homandberg 1999). Fibronectin levels in the model are directly influenced by a source parameter, $F_{dam}$, and a sink parameter, $\gamma _f$.

**Fig. 9:**
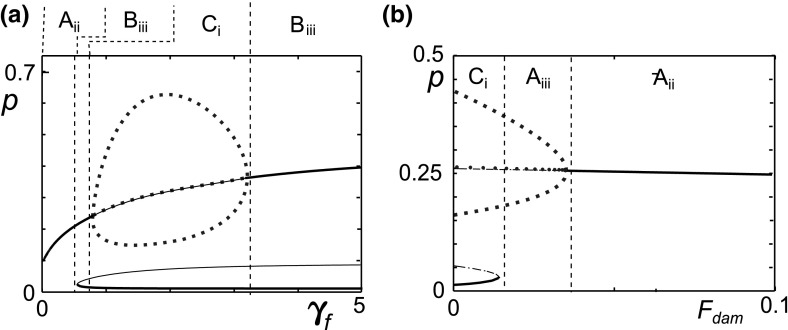
**a** Increased rates of fibronectin clearance, $\gamma _f$, can move the system from persistent inflammation, **b** increased damage, $F_{dam}$, has the opposite effect. Bifurcation plots of the pro-inflammatory cytokine level (*p*) against the mechanical damage and clearance parameters, $\gamma _f$ and $F_{dam}$. The *blue dotted lines* represent the minimum, maximum and average values of the limit cycles. The *dashed lines* denote the transition between different behaviours, which are labelled and summarised in Table 2 (colour figure online)

The bifurcation plot for $\gamma _f$, the clearance rate of Fn-fs (Fig. 9a), shows persistent inflammation for low $\gamma _f$ and bistability for higher $\gamma _f$. The bistable region is divided into an oscillatory inflamed state and a steady inflamed state. The value of *p* at the steady state lies within the range of the oscillatory behaviour so it is not clear which behaviour type would be most destructive to the cartilage. These bifurcations suggest that increasing Fn-fs clearance in an individual could be beneficial, possibly improving the outcome of treatment to reduce cytokine levels since increasing $\gamma _f$ can move the system from persistent inflammation to bistability. This is of particular interest since research has suggested that Fn-fs clearance may be affected by mechanical loading such as exercise (Evans and Quinn 2006; Zhang and Szeri 2005; O’Hara et al. 1990). Additionally, $\gamma _f$ principally represents removal of Fn-fs from the ECM via diffusion, which is likely to be reduced by the changes in the joint that are seen in OA, e.g. increased water content and immobility.

Mechanical damage is considered to be a major risk factor in OA and the first stage of repair after mechanical damage to the cartilage is necrosis of the damaged tissue which leads to increased concentrations of Fn-fs. We represent mechanical damage to the cartilage by an increase in the Fn-fs production rate, $F_{dam}$. Since $F_{dam}$ was zero in the reference parameter set, there is bistability at $F_{dam}= 0$ as before (Fig. 9b), which we might associate with the situation in normal cartilage. This behaviour has been observed in experimental work by Homandberg et al. (1997), who found that in normal cartilage tissue IL-6 levels became raised after treatment with high Fn-fs, and remained high over a period of 28 days. In contrast, for lower concentrations of Fn-fs the levels of IL-6 remained close to the control levels, suggesting a bistable system. As $F_{dam}$ increases, a fold bifurcation removes the lower two states leaving only persistent inflammation. This has not yet been shown experimentally. However repeating the experiments of Homandberg et al. (1997) with OA cartilage tissue may provide evidence of a behavioural change, and cytokine levels would be raised.

For the reference parameter set damage leads to sustained inflammation. However, as $F_{dam}$ increases we see that the level of *p* at the inflamed state reduces as does the level of *f*. These reduced levels still represent inflammation but may indicate slower disease progression. That an increase in $F_{dam}$ could slow disease progression is counter-intuitive and is likely to be due to dominance of the negative fragment-driven feedback.

### Other model parameters

**Fig. 10:**
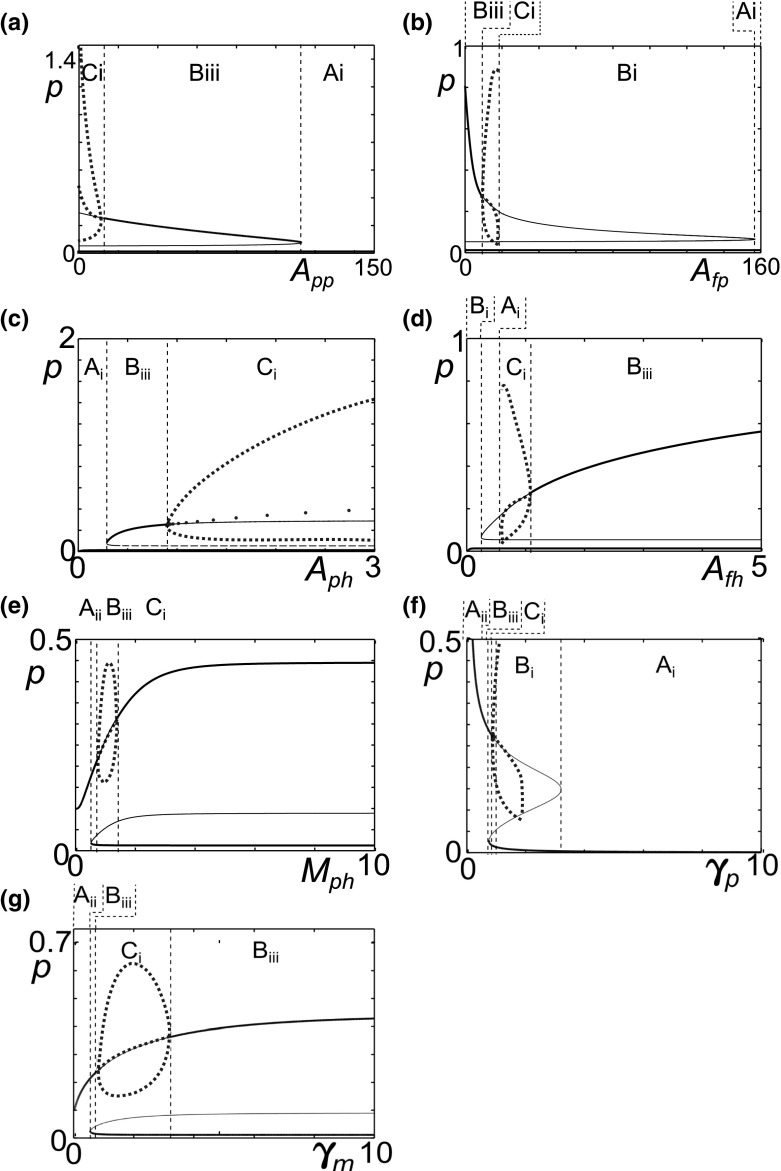
Model parameters go through bifurcations as their values are varied. Bifurcation plots of pro-inflammatory cytokine level (*p*) against parameters $A_{pp}, A_{fp}, A_{ph}, A_{fh}, M_{ph}, \gamma _p$ and $\gamma _m$. The *blue dotted lines* represent the minimum, maximum and average values of the limit cycles. The *black dashed lines* demarcate regions of different behaviour types listed in Table 2

Bifurcation analyses for the other model parameters are presented in Fig. 10. Anti-inflammatory cytokines reduce the production and activation of pro-inflammatory cytokines and hence we expect that higher values of $A_{pp}$ and $A_{fp}$, the anti-inflammatory cytokine production parameters, would lead to quiescence. Fig. 10a, b show that high levels of either parameter result in the loss of the inflamed state and a return to homoeostasis. This suggests that increases in anti-inflammatory cytokine production could be beneficial and lead to reduced or even halted cartilage degradation. Where there is an oscillatory inflamed state (Fig. 10b) decreases to anti-inflammatory production, move the system to a fixed inflammatory state at a lower value of *p* than the maximum of the inflamed limit cycle which may be beneficial depending on the relationship between cytokine level and cartilage destruction. Comparing Fig. 10a with the cytokine-only diagram (Fig. 5c), the bistable region occurs over a much wider range when we have fragment-driven dynamics. In some cases this could mean that inhibition of fragment-driven feedback could move the system from inflammation to quiescence.

The parameters $A_{ph}$ and $A_{fh}$ are the concentrations of *p* and *f* at which the anti-inflammatory cytokine production terms are half maximal. As such they represent the sensitivity of the anti-inflammatory cytokine response to pro-inflammatory cytokine and Fn-fs stimulation. When either $A_{ph}$ or $A_{fh}$ is small, the anti-inflammatory cytokine response is maximal at low inputs, and we only have a low single steady state, indicative of homoeostasis (Fig. 10c, d). At higher values of either $A_{ph}$ or $A_{fh}$, larger concentrations of *p* or *f* are required for anti-inflammatory cytokine production, and there is a fold bifurcation.

$M_{ph}$ governs the sensitivity of MMP production to activation by pro-inflammatory cytokine. When $M_{ph}$ is small there is a single stable steady state with a high value of *p* indicative of persistent inflammation (Fig. 10e). For higher values of $M_{ph}$ we move to bistability through a fold bifurcation, allowing the possibility of a move to a quiescent state. The inflamed state is oscillatory for some values of $M_{ph}$ due to two Hopf bifurcations.

Figure 10f shows that when the clearance of pro-inflammatory cytokines, $\gamma _p$, is low the steady state is at a high level of *p* indicating persistent inflammation as might be expected. When $\gamma _p$ is high there is a stable steady state, at low *p*, indicating homoeostasis. This implies that inactivation or rapid clearance of pro-inflammatory cytokines could be effective in halting the disease course of OA.

For this parameter set the bifurcation plots of $\gamma _m$ (Fig. 10g) are qualitatively similar to $\gamma _f$ (Fig. 9a) and show that as as $\gamma _m$ is increased the system moves from a persistent inflammation to bistability, with the possibility of a quiescent state.

### Tristable parameter values

**Fig. 11:**
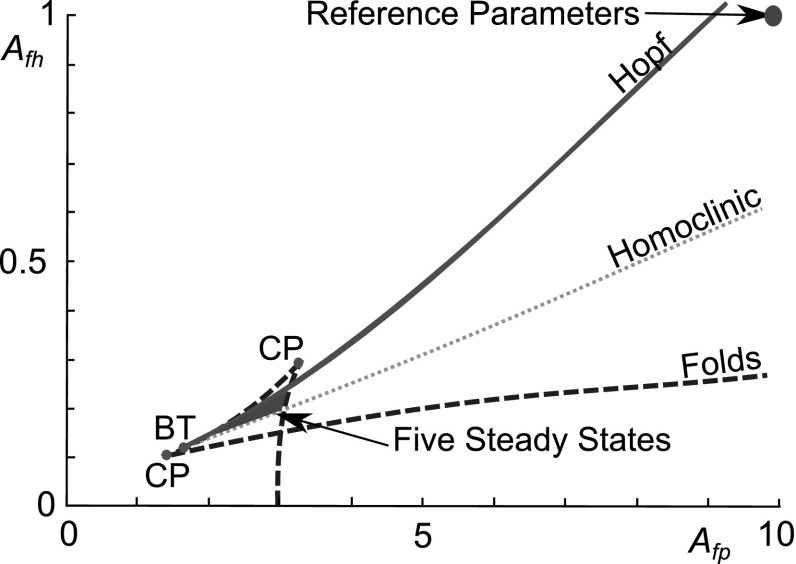
A small region with five steady states emerges as a result of Hopf and fold bifurcations. Two parameter bifurcation diagram showing curves of the Hopf and fold bifurcations in $A_{fp}$–$A_{fh}$ space, for the reference parameter set. The diagram shows that if we reduce the value of both $A_{fp}$ and $A_{fh}$ from the values of the reference set there is a region where there are five steady states as a result of fold and Hopf bifurcations (shown as dashed *blue* and *solid green lines* respectively). A homoclinic bifurcation is shown in *dotted black*, which emerges from a Bogdanov–Takens bifurcation (labelled BT). Cusp points are labelled CP (colour figure online)

Single parameter variations from the reference set showed at most 3 steady states, so we altered the parameter set to see if five-state behaviour emerged as is suggested by our analysis of the nullclines in Sect. 2. The local sensitivity analysis revealed that anti-inflammatory cytokine production parameters are the most sensitive. Figure 11 shows that five states emerge as a result of fold bifurcations as $A_{fp}$ and $A_{ph}$ are varied together. Where the Hopf and fold bifurcations meet there is a Bogdanov–Takens bifurcation point (Bogdanov 1981). From this point the homoclinic bifurcation emerges between the fold and Hopf bifurcations.

At $A_{fp}= 3.2$ and $A_{fh}= 0.2$ there are five steady states (Fig. 11), with two stable steady states and a stable limit cycle. As a result of the smaller value of $A_{fp}$ the maximum rate of anti-inflammatory cytokine feedback is lower than for the reference parameter set. However, a smaller $A_{fh}$ means the fragment-driven anti-inflammatory cytokine production may be stronger when levels of *f* are low. Tristability may be important for OA treatment since a move from one inflamed state to a less destructive one could slow the disease course where movement to a quiescent state is not possible. This will be discussed further in Sect. 4.

**Fig. 12:**
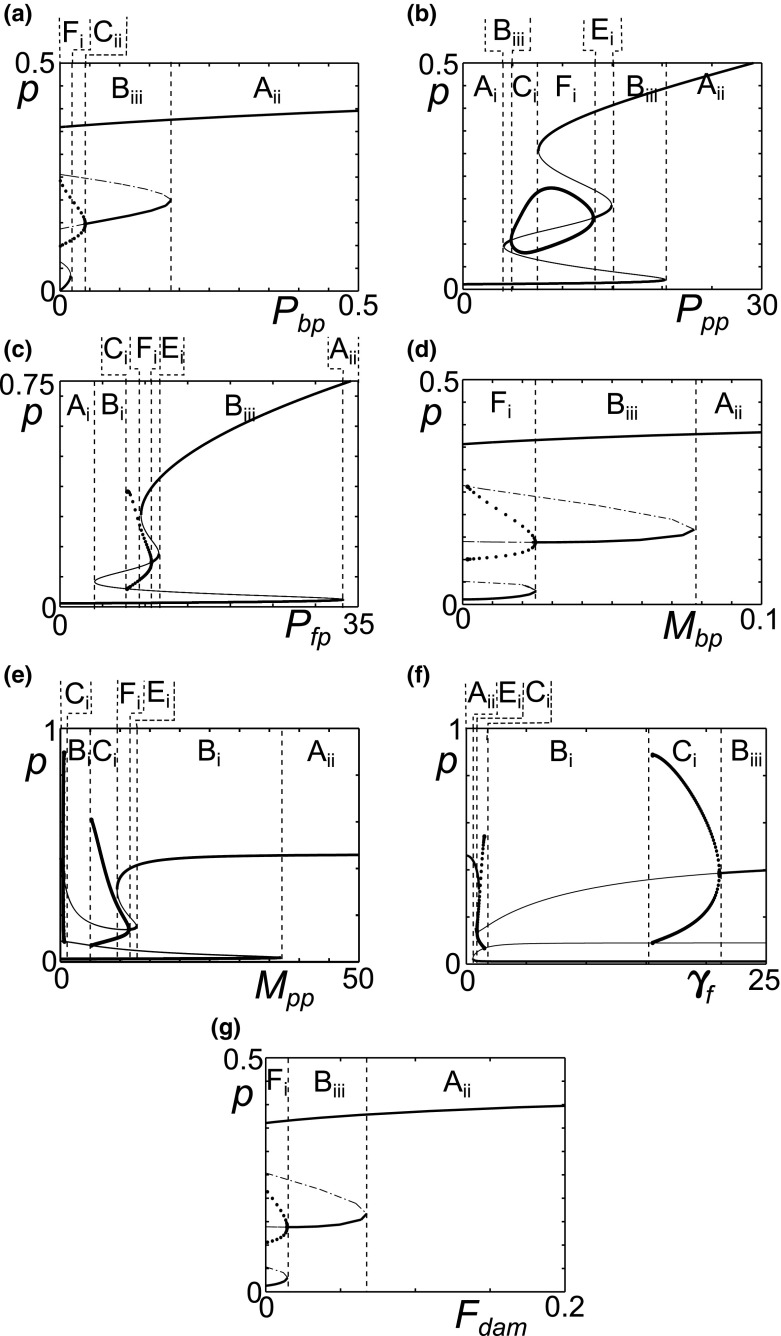
For some parameter ranges, one parameter variations show mono-, bi- and tri-stability. Bifurcation diagrams for Eqs. (5)–(8) in a parameter region with five steady states. Pro-inflammatory cytokine level plotted against **a** $P_{bp}$, **b** $P_{pp}$, **c** $P_{fp}$, **d** $M_{bp}$, **e** $M_{pp}$, **f** $\gamma _f$ and **g** $F_{dam}$. The *dashed lines* denote the transition between different behaviours, which are labelled and described in Table 2. The reference parameters, see Table 1 have been used except $A_{fp}=3.2$ and $A_{fh}=0.2$

Figure 12 shows bifurcation plots for single parameter variations of $P_{bp}, P_{pp}, P_{fp}, M_{bp}, M_{pp}, F_{dam}$ and $\gamma _f$ from this new parameter set. Comparing the plots in Fig. 12 to those of the reference parameter set (Fig. 6a), for $P_{bp}$ we see a reduced range of oscillatory behaviour and persistent inflammation occurs at lower values of $P_{bp}$. The bifurcation plot of $P_{pp}$, Fig. 12b, shows that homoeostasis emerges when $A_{fp}$ and $A_{fh}$ are lower, compared to Fig. 6b. As with $P_{bp}$ the range over which oscillatory inflammation occurs is much reduced. $P_{fp}$ variation (Fig. 12c) changes little from the bifurcations in the reference parameter set (Fig. 6c).

Bistability persists at high values of $M_{bp}$ when $A_{fp}$ and $A_{fh}$ are lower (Fig. 12d), however the levels of *p* in both states are high and suggest differing intensities of inflammation rather than quiescence and inflammation. The plot for $M_{pp}$ (Fig. 12e) compared to that of the reference parameter set (Fig. 7b), shows additional fold bifurcations in the upper branch. Additionally the limit cycle branches now collide with unstable branches at homoclinic bifurcation points. This has a significant effect as there is now a region of homoeostasis between two regions of bistability. This is also seen for $\gamma _f$. $F_{dam}$ (Fig. 12g), like $M_{bp}$, has a new region of bistability between two inflamed states.

### Robustness of behaviour groups

**Fig. 13:**
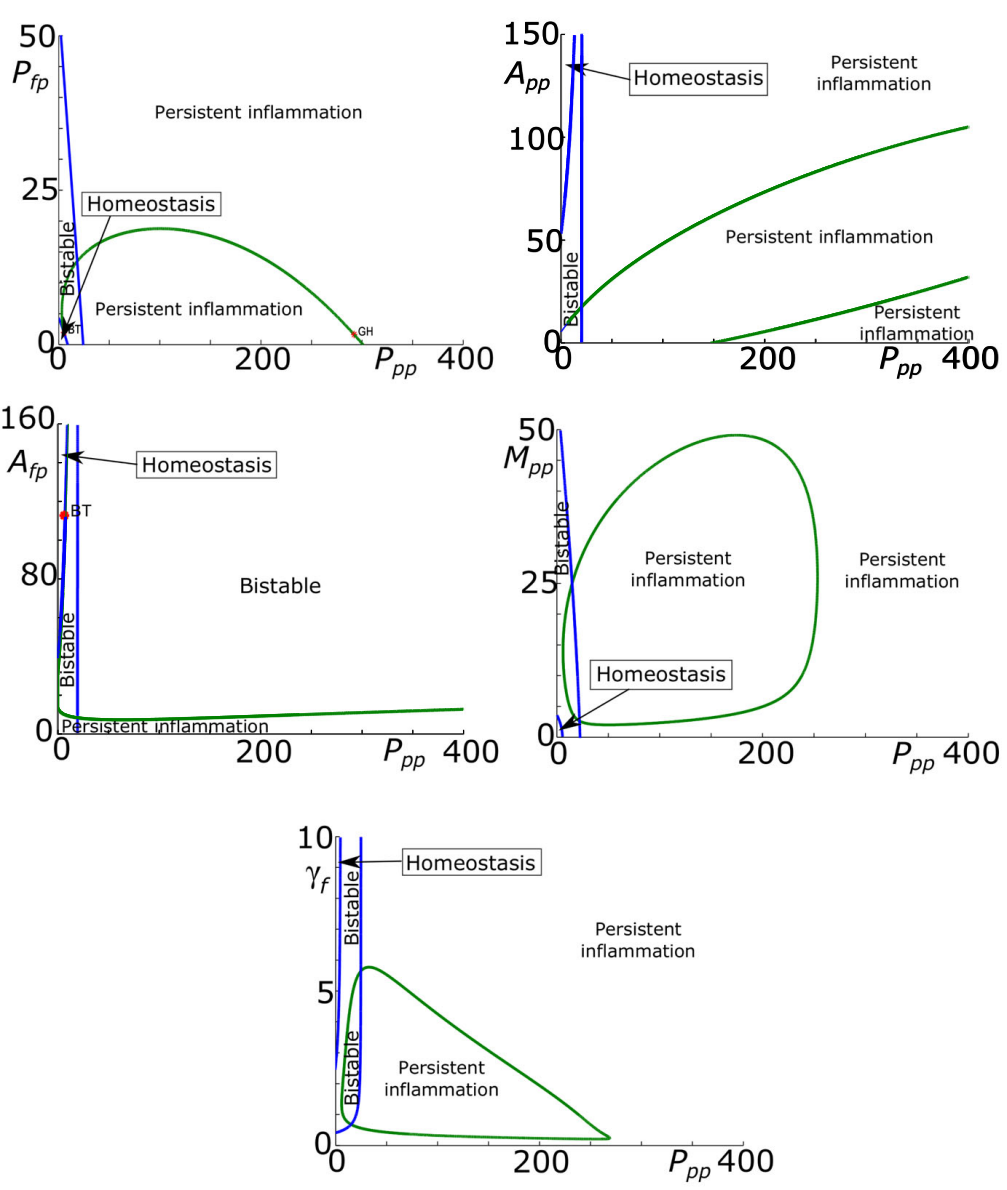
Two parameter bifurcation analysis showing robustness of the model [Eqs. (5)–(8)] behaviour to parameter variations. Two parameter bifurcation diagrams showing $P_{pp}$ against $P_{fp}, A_{pp}, A_{fp}, M_{pp}$ and $\gamma _f$. Fold bifurcations are shown as *blue lines* and Hopf bifurcations as *green lines*. Areas of homoeostasis, bistability and persistent inflammation are indicated. The reference parameters in Table 1 have been used (colour figure online)

Our analysis so far of one- and two-parameter variations has revealed behaviours that fall into four groups: homoeostasis, bistability, tristability and persistent inflammation. The manifestation of OA in individual patients may be determined by the specific behaviour type within one of the four groups, for example persistent inflammation that is steady or oscillatory. To further demonstrate robustness of the behaviour groups, in Fig. 13, we show additional two-parameter bifurcation studies for $P_{pp}$ varying along with each of $P_{fp},A_{pp}, A_{fp}, M_{pp}$ and $\gamma _f$. When $P_{pp}$ is low we generally have bistability, with disease occurring at higher levels of $P_{pp}$, so that this parameter is likely to be important in OA initiation and progression,regardless of the other parameters.

As in Fig. 11, we again see a co-dimension 2 Bogdanov–Takens bifurcation, at which fold and Hopf bifurcations collide. Close to this point, a limit cycle connects with one of the two steady states, resulting in the loss of the limit cycle to a homoclinic orbit (Izhikevich 2006). Often in the vicinity we also have a Generalised Hopf (GH) point (or Bautin bifurcation) where the Hopf switches from subcritical to supercritical. These are an important feature of the system as they change the nature of persistent disease from fixed to oscillatory or vice versa. There is no clinical evidence of oscillatory behaviour in OA, however, anecdotal evidence of patient experience (Hawker et al. 2008; Allen et al. 2009) suggests this is a possibility. We include two parameter bifurcation plots for the other combination of the six parameters in Fig. 13 in “Appendix 2”.

All of these bifurcation analyses demonstrate that the four key types of behaviour (homoeostasis, persistent inflammation, bistability and tristability) are robust core features of the system. In the next section we will consider possible treatment strategies for each of these behaviour groups.

## Treatment strategies

Clinical trials of disease modifying drugs for OA have not shown a slowing down of disease progression, as measured by pain, inflammation and joint space, or have had unexpected complications. Here we consider two of the main treatments that have reached clinical trials, anti-cytokine drugs and MMP inhibitors, as well as a further theoretical treatment possibility Fn-fs inhibition. Anti-cytokine therapy, licensed for use in RA, inhibits either the production or functioning of pro-inflammatory cytokines, usually TNF-$\alpha$. We model this as an instantaneous reduction in the level of pro-inflammatory cytokine, *p*. Additionally we consider an anti-cytokine therapy which instantaneously increases the level of anti-inflammatory cytokine, *a*. This type of treatment is licensed for RA but trials for OA have been unsuccessful (Chevalier et al. 2009). We model the effect of MMP inhibitors as an instantaneous reduction of *m*. In addition to these three types of therapies we also consider the possibility of Fn-fs as a target for OA treatment and model this as a reduction in the level of *f*. Here we consider how persistent inflammation, bistable and tristable behaviours may respond to these treatments.

### Treatment for bistable cases

In the bistable case (as in the reference parameter set we consider here) it is theoretically possible for an individual in an inflamed state to be moved to a state of quiescence, and this should be the aim for disease-modifying treatment, to achieve the best clinical outcome.

**Fig. 14:**
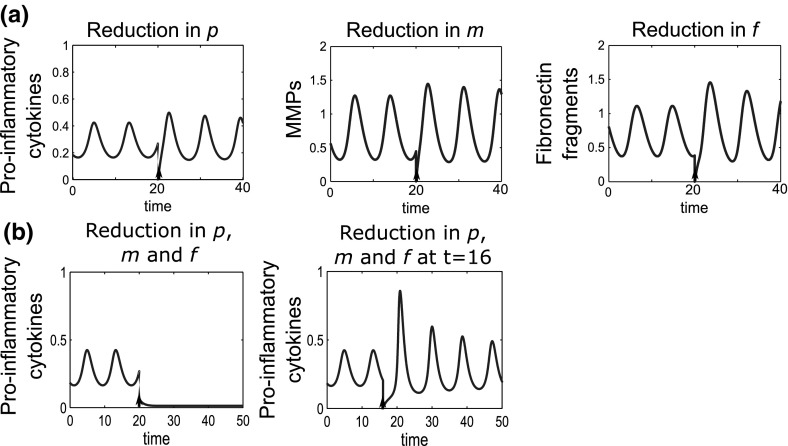
Monotherapy treatments are unable to move the system to quiescence, but combined treatments can be effective. **a** Time course simulations of single treatments where the system displays bistable behaviour. At $t=0$ the system is at the disease limit cycle. A single dose of anti-cytokine (reduction in *p*), MMP inhibition (reduction in *m*) or Fn-fs inhibition (reduction in *f*) treatment was simulated at $t=20$. The dose size given in each case was the maximum possible (i.e. an instantaneous decrease to zero of each of the variables). **b** Time course simulations of combined treatments where we have bistable behaviour in the system. A single combined dose of anti-cytokine, MMP inhibition and Fn-fs inhibition treatment was simulated at $t=20$ and $t=16$. The dose size is the minimum dose size (see text) that moves the system to health [0.2(*p*), 0.5(*m*) and 0.4(*f*)]. The diagrams show that dose timing as well as dose size is important. The reference parameter set was used for these simulations

In this model, for the reference parameter set, we tried single doses of anti-cytokine therapy, MMP inhibition and Fn-fs inhibition. In each case we simulated the effect of applying the largest possible dose by reducing the level to zero, but none of these treatments moved the system to quiescence (Fig. 14a), since the system was not moved outside the basin of attraction of inflammation. This result is in line with data from clinical trials of anti-cytokine and MMP inhibition treatments, that have shown no long term benefit in single dose therapy (Qvist et al. 2008).

We have found that a combined treatment strategy can bring the system to a state of quiescence. Over several simulations we varied the timing and reduced the magnitude of the doses used, until we found the smallest dose of each treatment that would move the system from inflammation to quiescence when all three treatments were combined (Fig. 14b). In this case the magnitudes of the variable reductions are 0.2(*p*), 0.5(*m*) and 0.4(*f*).

Timing of the dose is of crucial importance, particularly for this parameter set since the inflamed state is oscillatory. If a dose is given at the wrong point in the limit cycle then it may not be large enough to move out of the basin of attraction of the inflamed state and may result in a period of increased amplitude oscillations as it moves back to the inflamed state (Fig. 14b). This type of behaviour could have large implications both for clinical trial results and treatment regimens for drugs taken to market.

**Fig. 15:**
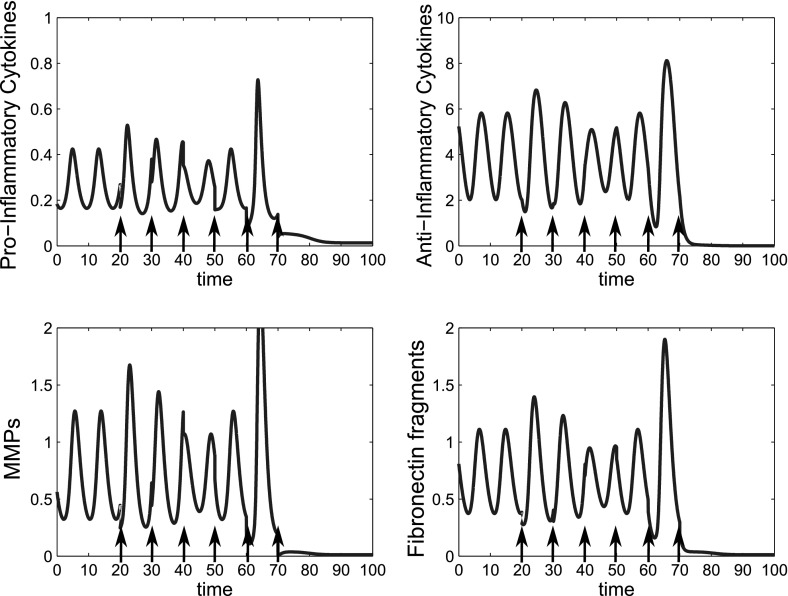
Multiple doses of combined therapy allow the individual dose magnitude to be reduced. Time course simulations of multiple combined treatments where we have bistable behaviour in the system. At $t=0$ the system is at the inflamed limit cycle. Six combined doses of anti-cytokine, MMP inhibition and fibronectin fragment inhibition treatment are simulated starting at $t=20$, with a dose interval of ten time units. The dose magnitude for each of the six doses is 0.1(*p*), 0.2(*m*) and 0.1(*f*). The reference parameter set was used for these simulations

We find that multiple doses of treatment given over time can also reduce the system to quiescence and allow smaller individual doses to be given. Figure 15 shows a series of six doses, given ten time units apart, which moves the system to quiescence; the magnitude of each dose is 0.1(*p*), 0.2(*m*) and 0.1(*f*). Compared to the single dose strategy this represents a large reduction in dosage at any particular time. This may be beneficial if there are side effects associated with the drugs, although overall, the total amount of drugs given would be higher in this case. As with the single dose, the timing and size of the dose is important as well as the total number of doses.

**Fig. 16:**
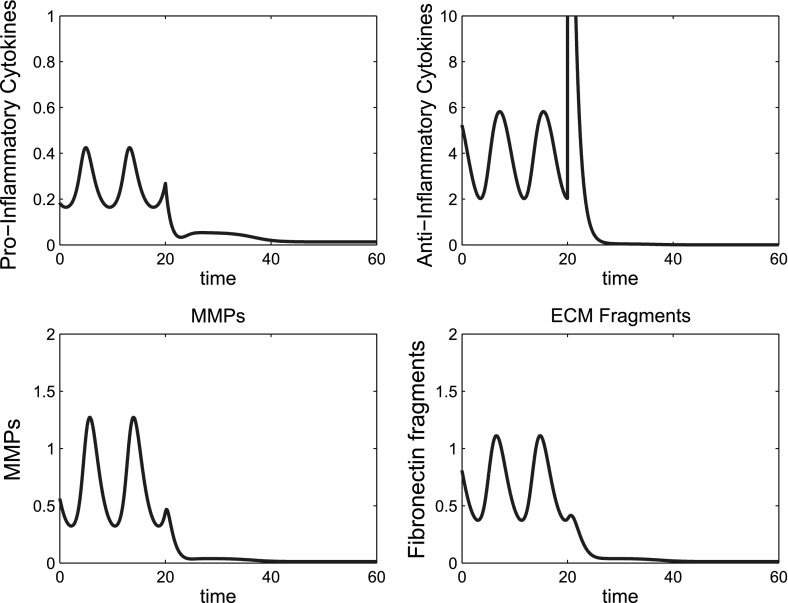
A high dose of anti-inflammatory cytokines can bring the bistable system to the quiescent state. Time course simulations of single doses of anti-inflammatory cytokines where we have bistable behaviour in the system. At $t=0$ the system is at the inflamed limit cycle. A dose of 40 units of *a* is given at t $=$ 20 bringing the system to quiescence. The reference parameter set (Table 1) was used for these simulations

Anti-inflammatory cytokines are not currently used in anti-cytokine therapy as they have had poor joint response in clinical trials. We found that a single dose of anti-inflammatory cytokines was able to bring the system to a quiescent state from the inflamed state. Figure 16 shows that a dose of 40 units moves the system to quiescence, when given at t $=$ 20. This dose is the lowest that will bring the system to quiescence. However, this dose is an order of magnitude greater than the anti-inflammatory cytokine level at the inflamed state, so may not be clinically feasible.

Dose timing is not trivial; the optimal timing is not at the highest point of *p* of the limit cycle as might be expected, but at the point where the system is closest to the basin of attraction of the quiescent state. This point may vary between individuals so individually tailored treatment plans may be necessary for most effective treatment.

**Fig. 17:**
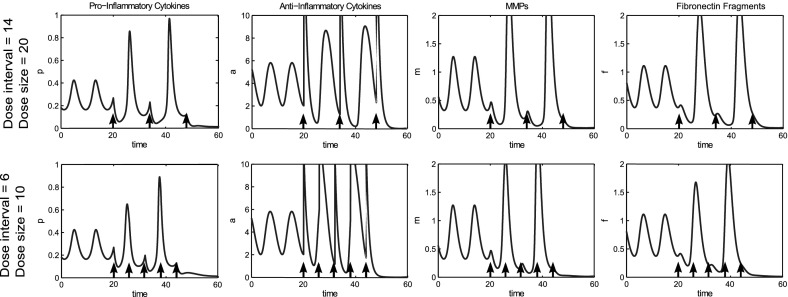
Multiple smaller doses of anti-inflammatory cytokine can move the system to quiescence but dose timing, interval and size are all crucial to treatment outcome. Time course simulations of multiple doses of anti-inflammatory cytokines where we have bistable behaviour in the system. At $t=0$ the system is at the disease limit cycle. In the *top row* three doses of 20 units of *a* are given as indicated by the *black arrows*. In the *bottom row* five doses of 10 units of *a* are given as indicated by *black arrows*. The reference parameter set (Table 1) was used for these simulations

We investigated application of multiple doses of anti-inflammatory cytokines in order to reduce the dose size necessary. By giving 3 doses at intervals of 14 time units starting at t $=$ 20 we were able to bring the system to quiescence with a dose size of 20, reduced from 40 in the single dose case at this time (Fig. 17). Again timing of the initial dose,the dose interval and number of doses are of crucial importance.

**Fig. 18:**
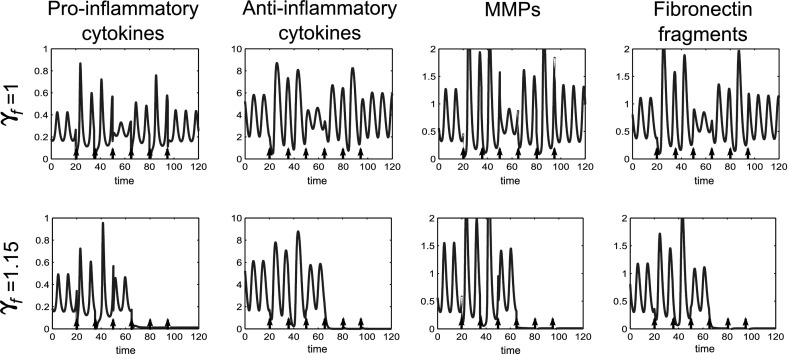
An increased rate of Fn-fs clearance can replace Fn-fs inhibition therapy in combined treatment regimes. Time course simulations of multiple combined treatments where the system displays bistable behaviour. The *first row* shows the system with the reference parameter set, whilst the *second row* shows the same parameters except that $\gamma _f$ is increased by 15%. At $t=0$ the system is at an inflamed limit cycle. Six combined doses of only anti-cytokine and MMP inhibition treatment are simulated starting at $t=20$, with a dose interval of ten time units. The dose magnitude for each of the six doses is 0.4(*p*) and 0.4(*m*)

Finally for the bistable case we have considered how an increased rate of Fn-fs clearance could affect treatment options. Research has shown that clearance of macromolecules such as Fn-fs is increased in the cartilage with cyclic loading (Evans and Quinn 2006), so an increase in $\gamma _f$ alongside reductions in *p* or *m*, may be representative of a course of exercise or physiotherapy in combination with disease-modifying drugs. Increasing the value of $\gamma _f$ has a similar effect to Fn-fs inhibition and simulations show that if this is raised we no longer need to alter the amount of *f* to bring the system to quiescence (Fig. 18). This may mean that combined anti-cytokine and MMP inhibition therapy, alongside physical therapy, could be a viable treatment option.

### Treatment for tristable cases

**Fig. 19:**
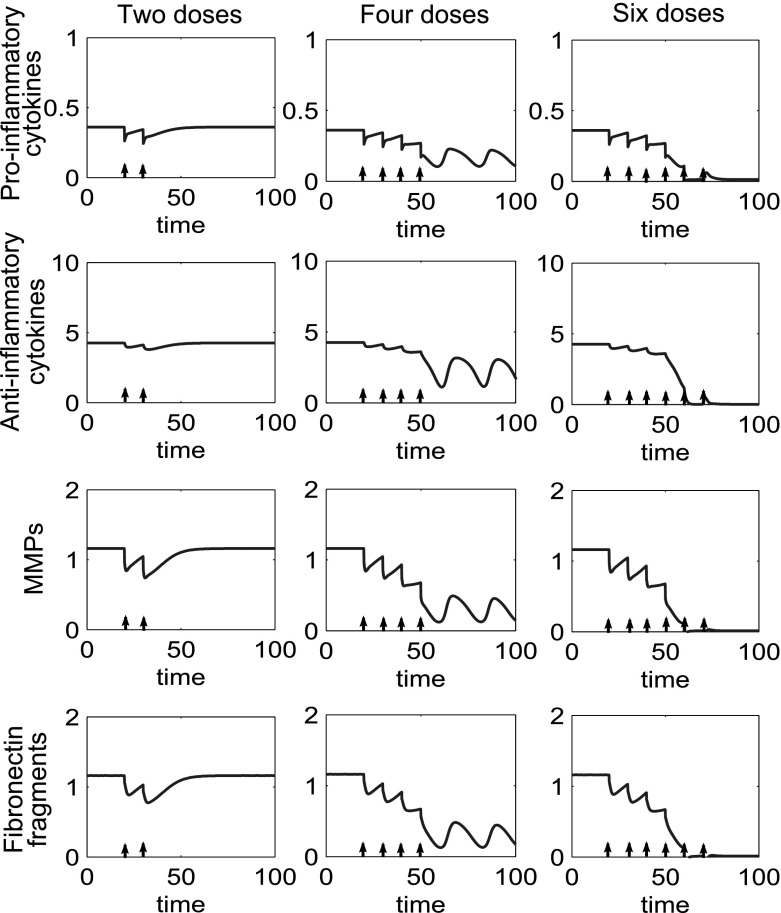
In the tristable system multiple doses may move the system to quiescence and a reduced number of doses may move the system to a less inflamed state. Time course simulations of multiple combined treatments where the system displays tristable behaviour. The *first column* shows two doses of treatment, the *second column* four doses and the *third column* six doses. The doses of anti-cytokine, MMP inhibition and fibronectin fragment inhibition treatment are simulated starting at $t=20$, with a dose interval of ten time units. The dose magnitude for each dose is 0.1(*p*), 0.2(*m*) and 0.1(*f*)

Where we have tristable behaviour we generally have two inflamed states and one quiescent state. Simulations of treatment options for this type of behaviour show that if the system is at either one of the inflamed states it will act as in the bistable case and can be moved to the quiescent state, with a sufficient number of doses of combined treatments. Additionally if the system is at the higher inflamed state it can be moved to the lower inflamed state with fewer doses of treatment than are required to move the system to quiescence. Figure 19 shows multiple doses of combined treatments of anti-cytokine, MMP inhibition and Fn-fs clearance therapies. Where two doses are given the system returns to the original inflamed state. When four doses are given the system moves to a lower inflammatory state, which in this case is a limit cycle. Six doses are sufficient to move the system to a state of quiescence.

**Fig. 20:**
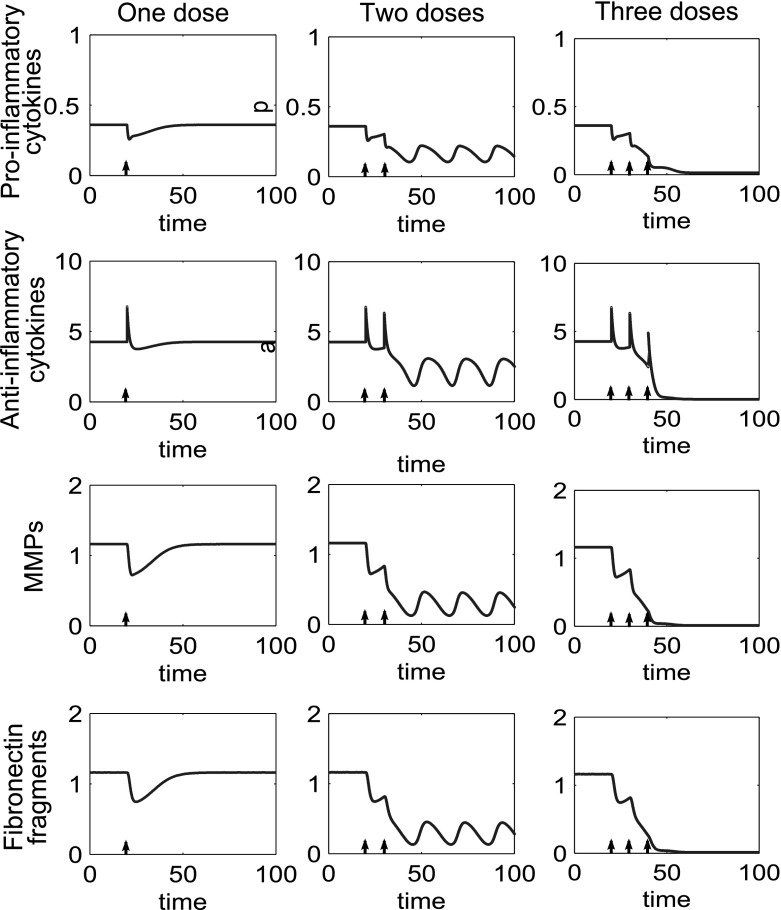
In the tristable system multiple doses of anti-inflammatory cytokine bring the system to either a lower inflamed state or quiescence depending upon the number of doses given. Time course simulations of multiple doses of anti-inflammatory cytokine where we have tristable behaviour in the system. The *first column* shows one dose of treatment, the *second column* two doses and the third column three doses. The doses of anti-inflammatory cytokine have a magnitude of 2.5 and are simulated starting at $t=20$, with a dose interval of ten time units. The number of doses determines which state the system is moved to

Figure 20, shows a similar pattern of behaviour for anti-inflammatory cytokine therapy. For this parameter set much lower doses of *a* bring about quiescent compared to the bistable case.

### Treatment for persistent inflammation

**Fig. 21:**
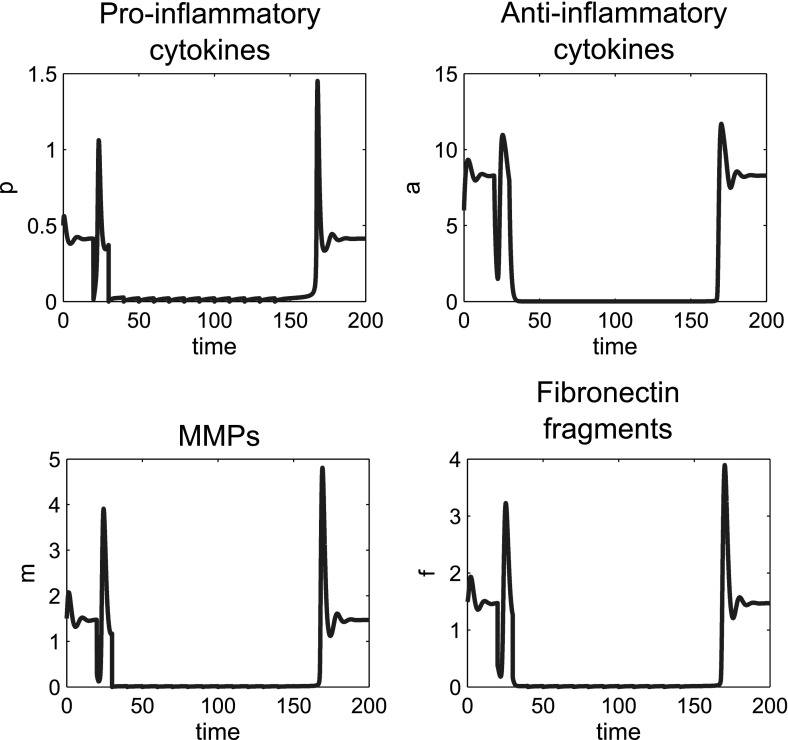
With persistent inflammation combined therapy can induce quiescence but cessation of treatment would allow inflammation to return. Time course for the system showing persistent inflammation. Multiple combined treatments are given ten time units apart starting at time 20. The dose size for the treatments are 0.4(*p*), 1.2(*m*) and 1.1(*f*) representing a $98\%(p), 82\%(m)$ and $75\%(f)$ reduction from the inflamed state. These lower the system variables to a quiescent level. Parameters used are the reference parameter set as described in Sect. 3, except for $P_{fp}=40$

In cases of persistent inflammation moving to a quiescent state is not possible without parameter changes. However, disease control may still be possible with ongoing doses of disease modifying drugs (Fig. 21) which can reduce the cytokine and fibronectin levels to quiescent levels, although this is not self-sustaining. In this case the dose size required is much higher than that needed in the bistable case, to bring the system to low cytokine levels.

There is a trade off between dose size and dose interval, with smaller intervals allowing a decrease in the size of the dose. However, in clinical practice there would be both medical and practical considerations in reducing dose interval.

Repeated doses of anti-inflammatory cytokine can also reduce the cytokine and fibronectin levels. However, the dose size needs to be very high. For this parameter set a dose size of 500 units, 62 times greater than the inflamed state is required. The system is also very sensitive to dose size with some smaller dose sizes increasing the levels of *p*. The large dose size and sensitivity to change may make this type of treatment difficult to implement in a clinical setting.

## Discussion

Osteoarthritis is associated with both clinical and sub-clinical inflammation (Hoff et al. 2013; Wojdasiewicz et al. 2014). It is now largely accepted that cytokines, MMPs and fibronectin fragments are key inflammatory mediators in destructive OA mechanisms. Effective disease-modifying therapies are much needed for OA (Qvist et al. 2008; Conaghan 2013), and these pathways appear to offer good targets. However, the lack of success in clinical trials suggests that we may not understand sufficiently well the dynamic interactions of these pathways. To gain a better understanding of the nature of these dynamics we grouped cytokines by function, either pro-inflammatory or anti-inflammatory, in addition to MMPs and Fn-fs, simplifying the problem to four variables.

Inflammatory cytokines may be raised in osteoarthritis as a result of loss of ECM homoeostasis (Creamer and Hochberg 1997). Possible causes of this include increased numbers of cytokine receptors on chondrocytes, making them more sensitive to up-regulation (Wojdasiewicz et al. 2014) and over expression of either cytokines or MMPs as a result of secretory cell senescence where cells over produce cytokines as the result of genomic damage in aging chondrocytes (Berenbaum 2013). Regardless of cause the net result is a loss of the homoeostatic balance of ECM turnover. Bifurcation analysis of the model revealed a range of behaviour types, which in general terms we class as homoeostasis, persistent inflammation, bistable inflammation or tristable inflammation. Individuals in the homoeostasis group would not be susceptible to developing OA, whereas those in the other groups would either be susceptible or have developed OA. In the cytokine-only model for rheumatoid arthritis (Baker et al. 2013), the regions of homoeostasis were relatively large and we suggested that many individuals would, therefore, not be susceptible to RA. However in the OA model presented here, in the parameter ranges we considered, homoeostasis only accounted for very small regions in comparison with the other behaviours (Figs. 6, 7, 9, 10, 13). This suggests that most individuals will be susceptible to developing OA, given an appropriate stimulus. Ageing chondrocytes, and indeed increasing frailty with aging, increase the likelihood of such a stimulus being sufficient to lead to OA changes. This corresponds with the aetiology of OA, given the large percentage of affected individuals including asymptomatic individuals, and the strong association with age.

In the bulk of this paper we have focused on equal Hill coefficients with $n=2$ for all regulatory functions. There is no experimental evidence to indicate what *n* should be, or indeed whether all the coefficients should be equal. For equal Hill coefficients greater than 2 we have very similar qualitative behaviour to $n=2$, except for very small regions of parameter space we have a greater number of steady states. This can also be the case with mixed coefficients greater than 2. For example, we have been able to find up to seven steady states with mixed Hill coefficients of 2 and 4. The only major behavioural change in the system comes when all $n = 1$, giving a single steady state which would represent homoeostasis or persistent inflammation, depending on the value of *p* (shown in “Appendix 1”). There would be no bifurcation behaviour in the model and treatment in this case would be as discussed in Sect. 4.3. We have no reason to believe that the special case of all $n=1$ is likely. Our variables are not single signalling molecules but functional classes and, given the redundancy in cytokine signalling mechanisms many of these are likely to be up-regulated in OA simultaneously. Furthermore, the Hill functions represent pathways involving multiple receptors, and transcriptional regulations which are likely to involve multiple transcription factors per signalling protein (Begitt et al. 2014). Therefore, it seems entirely feasible that the case with $n\ge 2$ is representative of the in vivo situation.

Both oscillatory and steady inflammatory states are present in the model. In the steady case it is likely that such patients would see a gradual increase in inflammation and disability as a result of consistently high cytokine levels. OA progression is generally assessed through patient pain score and decreasing mobility. Some OA patients report intermittent periods of pain in early OA and a pattern of *flare ups* in OA is commonly reported (Creamer and Hochberg 1997; Hawker et al. 2008; Maly and Cott 2009; Allen et al. 2009), which may be a manifestation of the limit cycles we see in the model, or movement between quiescent and inflamed states as levels of cytokines, MMPs or Fn-fs fluctuate. This pattern of behaviour in RA has been linked to cyclic levels of cytokines, and the same may be true for OA, although data is not yet available for OA. The treatments we apply in this model move the system away from a disease state, and these treatments will work both in the case of fixed and oscillatory inflammation. It may be possible, with disease modifying drugs, to move a patient from an oscillatory inflamed state to a fixed inflammatory state, such as in the tristable case illustrated in Fig. 20. In this model, we do not examine which type of inflammatory behaviour is most destructive long term, but it may be possible to explore this with a spatial model of OA cartilage.

Examination of current research and clinical practice gives insight into potential treatment strategies. Currently no disease-modifying treatments are available for OA. Amongst those undergoing clinical review are anti-cytokine and MMP inhibitor therapies. We have considered four different treatment strategies: anti-cytokine therapy, anti-inflammatory cytokines, MMP inhibitors and Fn-fs inhibitors. We found a combined treatment strategy to be the most effective at treating bistable, tristable and persistent inflammation. Our model predictions further suggest dose size and timing are important to treatment outcome and it may be possible to optimise these using control theory. We found that the only effective monotherapy was to use anti-inflammatory cytokines, although this treatment requires very high dose sizes relative to steady state concentrations. Treatment outcome is predicted to be highly sensitive to dose timing and interval since the basin of attraction of the quiescent state is small and local to the state. These issues may make clinical treatment with anti-inflammatory cytokines, such as IL-1Ra, infeasible and may explain the failure of IL-1Ra drugs trials (Chevalier et al. 2009), despite promising experimental results which showed the response to anti-inflammatory therapy in animal models (Caron et al. 1996; Fernandes et al. 1999). For the other three treatment options any one of these alone is predicted to be ineffective, and combined treatments seem to be necessary (Sect. 4). We can see from the phase diagram (Fig. 3) why this is the case. Any move from the inflamed state in only one direction would remain in the basin of attraction of the inflamed state. In all the parameter sets that we considered the basin of attraction of the quiescent state was small. Whilst our theoretical model is qualitative in nature, the results suggest that combined treatments offer a much better possibility of success than single treatments, even where the single treatment shows no benefit alone (Fig. 14). We saw that in the case of tristability we had an option of treatment to move the system from a higher inflamed state to that of a lower one. If there were additional inflamed states, as considered in Sect. 2, it may be possible, in principle, to move an individual from the higher inflamed state to any of these lower states. However it is unlikely that in the foreseeable future we would be able to identify individuals with such multiple inflamed states or personalise the treatment plan to such a degree.

Some evidence of the feasibility the treatments we have suggested may come from studies into joint lavage therapy. Although controversial due to mixed results, this treatment involves *flushing* an OA joint with saline which is analogous to reducing the value of variables in the model. Some studies have reported relatively long term improvements in patients given this treatment (Arden et al. 2008). The varied results of such treatments with some patients finding it ineffective, corroborates the likely existence of different disease phenotypes.

Clinical and experimental data could provide validation for this theoretical model. Lack of data and the difficulty of obtaining data relating to cytokine and MMP dynamics in OA means that the existence of the behaviour dynamics found in this model has not yet been proven in a clinical or experimental setting. However, studies of cytokine and MMP expression in OA have shown that these are raised both in early and advanced OA (Scanzello et al. 2009). This suggests the presence of an inflamed state where levels of cytokines and MMPs are raised, in comparison to a quiescent state where they remain low as predicted by our model. The study of Homandberg et al. (1997) indicates that bistability exists within normal human cartilage, and could be extended to investigate whether this changes in osteoarthritic cartilage. More experimental work would need to be conducted in this area to show the difference between early and late stage OA, which would help identify which type of treatments would be appropriate. However, since the structure of the cytokine network we model here is well established (Goldring 2000a; Martel-Pelletier et al. 1999; Tetlow et al. 2001; Sandell and Aigner 2001) we are confident that such states could exist, and hence that treatments of the types we have theorised are realistic experimental targets. The lack of biomarkers (easily measurable indicators of disease severity) for OA makes oscillatory behaviour difficult to track, but periods of *flare up* are widely reported anecdotally and considered part of the disease course, particularly in early OA. Associations between these periods of OA flare up, increased MMP expression and increased cartilage degradation have been reported (Manicourt et al. 2005).

Limitations of the model include the lack of a link to clinical disease measures, which include joint space narrowing and radiographic evidence of cartilage deterioration. As better measures of OA disease activity are developed, such as OA biomarkers we may be able to draw more detailed conclusions about OA disease dynamics in future studies. We have not explored spatial and mechanical aspects of the disease, which play a large role in OA progression, and have been explored mathematically by others. In this paper we have explored the behaviour of cytokine interactions in the joint and identified potential areas of future research into OA treatment strategies. Although we have been unable to directly validate the model due to a lack of quantitative, comparable experimental or clinical data, we have found that the model mimics many clinical features of the condition. We believe that future work in this area needs to combine all these aspects of OA and joint mechanics, as it is becoming increasingly clear from biological research that the interactions between physical and biochemical factors in OA are significant.

## Figures and Tables

**Fig. 22:**
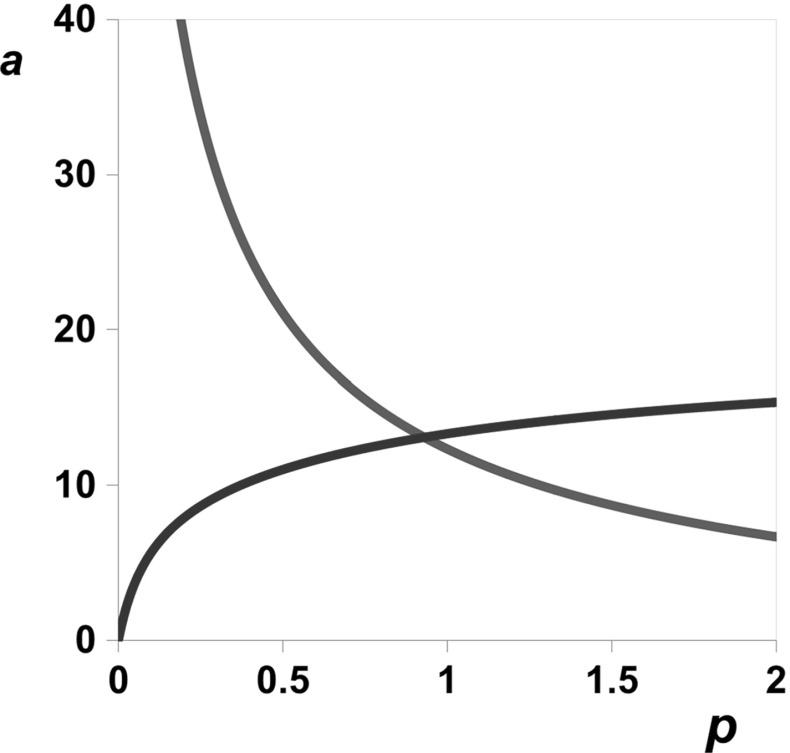
Forms of the *p* **and** *a* nullclines when the Hill coefficients $=$ 1. The *a*-nullcline is monotonically increasing whilst the *p*-nullcline is monotonically decreasing leading to only one possible steady state which is stable

**Fig. 23:**
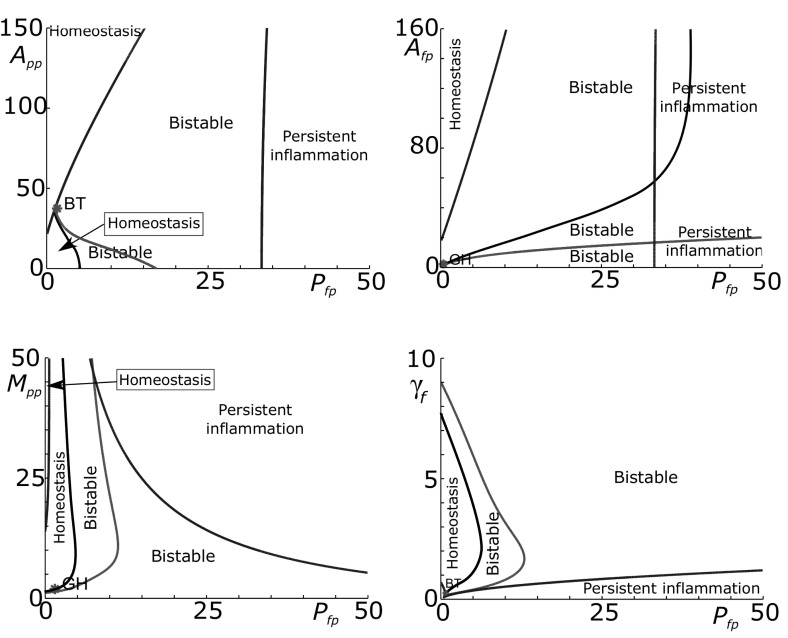
Two parameter bifurcation diagrams showing $P_{fp}$ against $A_{pp}, A_{fp}, M_{pp}$ and $\gamma _f$. Fold bifurcations are shown as *blue lines*, Hopf bifurcations as *green lines* and homoclinic bifurcations are shown in *black*. Areas of monostable health, bistability and monostable disease are indicated. The reference parameter set is used (colour figure online)

**Fig. 24:**
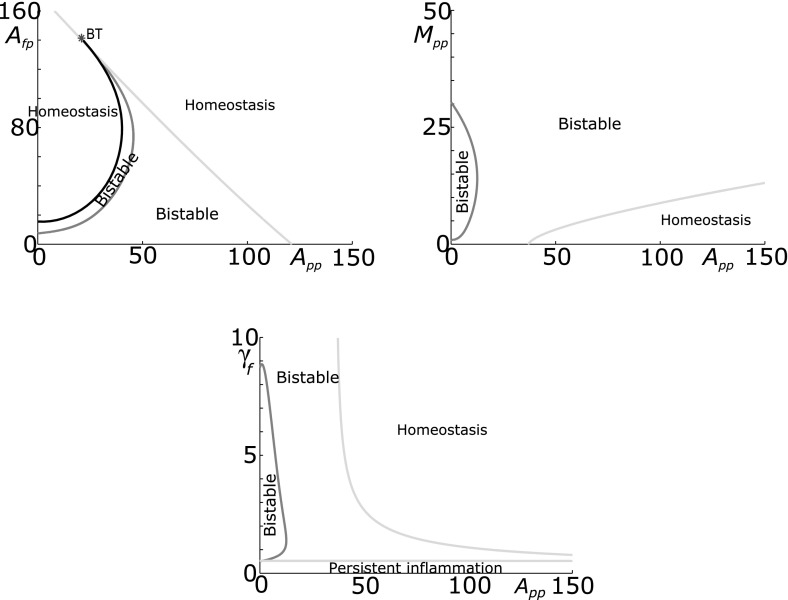
Two parameter bifurcation diagrams showing $A_{pp}$ against $A_{fp}, M_{pp}$ and $\gamma _f$. Fold bifurcations are shown as *blue lines* and Hopf bifurcations as *green lines*. Areas of monostable health, bistability and monostable disease are indicated. The reference parameter set is used (colour figure online)

**Fig. 25:**
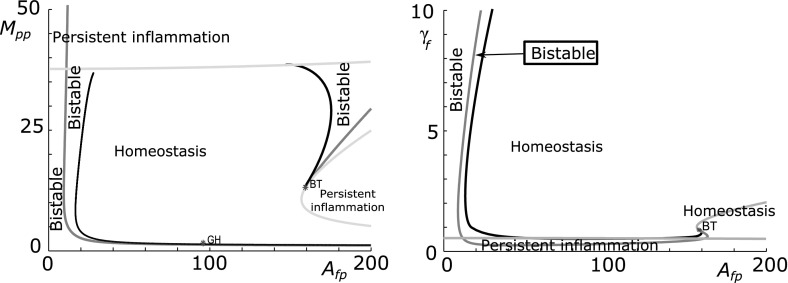
Two parameter bifurcation diagrams showing $A_{fp}$ against $M_{pp}$ and $\gamma _f$. Fold bifurcations are shown as *blue lines*, Hopf bifurcations as *green lines* and homoclinic bifurcations are shown in *black*. Areas of monostable health, bistability and monostable disease are indicated. The reference parameter set is used (colour figure online)

**Fig. 26:**
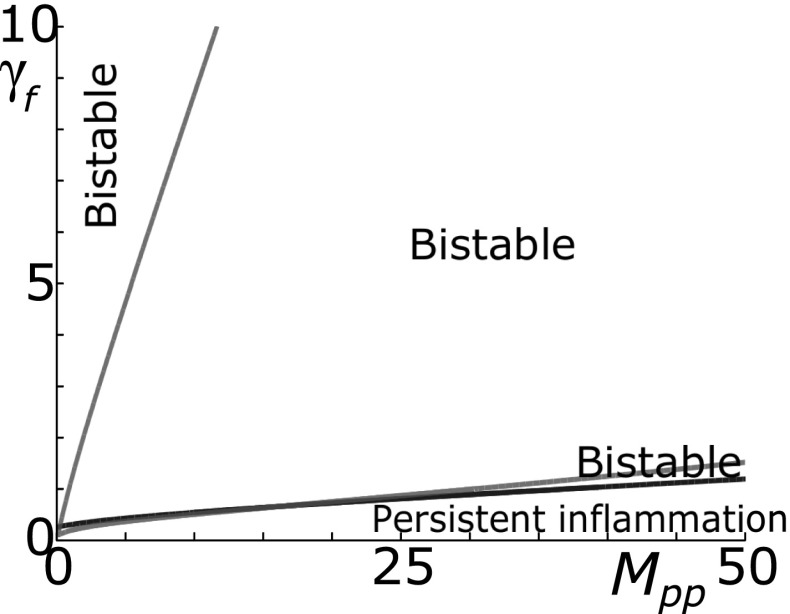
Two parameter bifurcation diagram showing $M_{pp}$ against $\gamma _f$. Fold bifurcation is shown as a *blue line* and Hopf bifurcations as *green lines*. Areas of bistability and monostable disease are indicated. The reference parameter set is used (colour figure online)

## Appendix 1: Hill coefficients $=$ 1

When the Hill coefficients are all 1 the dimensionless model simplifies to

20 $$\begin{aligned} \frac{\mathrm{d} p}{\mathrm{d} t}&= \left( P_{bp}+ P_{pp}\frac{p}{1 + p} + P_{fp}\frac{f}{1+f}\right) \left( \frac{1}{1 + a} \right) - \gamma _p p \end{aligned}$$

21 $$\begin{aligned} \frac{\mathrm{d} a}{\mathrm{d} t}&= A_{pp}\frac{p}{{A_{ph}}+p} + A_{fp}\frac{f}{A_{fh}+f} - a \end{aligned}$$

22 $$\begin{aligned} \frac{\mathrm{d} m}{\mathrm{d} t}&= M_{bp}+ M_{pp}\frac{p}{{M_{ph}} + p} - \gamma _m m \end{aligned}$$

23 $$\begin{aligned} \frac{\mathrm{d} f}{\mathrm{d} t}&= m + F_{dam}- \gamma _f f. \end{aligned}$$

At steady state we have

24 $$\begin{aligned} a&=\left( \frac{1}{\gamma _p p}\right) \nonumber \\&\quad \times \left( P_{bp}+ P_{pp}\frac{p}{1+p} + P_{fp}\frac{\left( F_{dam}\gamma _m + M_{bp}+ M_{pp}\frac{p}{M_{ph}+ p} \right) }{({\gamma _f \gamma _m})+\left( F_{dam}\gamma _m + M_{bp}+ M_{pp}\frac{p}{M_{ph}+ p} \right) }\right) -1 \end{aligned}$$

and

25 $$\begin{aligned} a&= A_{pp}\frac{p}{A_{ph}+p} \nonumber \\&\quad + A_{fp}\frac{\left( F_{dam}\gamma _m + M_{bp}+ M_{pp}\frac{p}{M_{ph}+ p} \right) }{(\gamma _f \gamma _m)A_{fh}+\left( F_{dam}\gamma _m + M_{bp}+ M_{pp}\frac{p}{M_{ph}+ p} \right) }. \end{aligned}$$

Given that all the parameters must be positive Eq. (24) must be monotonically decreasing and Eq. (25) must be monotonically increasing (Fig. 22). This allows for only one steady state, which is always stable. Hence, depending on the value of *p* at the steady state, we have either behaviour type $Ai$ (homoeostasis) or behaviour type $Aii$ (persistent inflammation). Treatments for persistent inflammation are discussed in Sect. 4.3.

## Appendix 2: Additional two parameter bifurcation diagrams

We show here further exploration of the global parameter space by looking at two-parameter bifurcation diagrams of combinations of parameters $P_{fp}, A_{pp}, A_{fp}, M_{pp}$ and $\gamma _f$ (Figs. 23, 24, 25, 26).

## References

[CR1] Allen K, Coffman C, Golightly YM, Stechuchak K, Keefe F (2009). Daily pain variations among patients with hand, hip, and knee osteoarthritis. Osteoarthr Cartil.

[CR2] Arden N, Cooper C (2005). Osteoarthr Handb.

[CR3] Arden N, Reading I, Jordan K, Thomas L, Platten H, Hassan A, Ledingham J (2008). A randomised controlled trial of tidal irrigation vs corticosteroid injection in knee osteoarthritis: the KIVIS study. Osteoarthr Cartil.

[CR4] Baker M, Denman-Johnson S, Brook BS, Gaywood I, Owen MR (2013). Mathematical modelling of cytokine-mediated inflammation in rheumatoid arthritis. Math Med Biol.

[CR5] Begitt A, Droescher M, Meyer T, Schmid CD, Baker M, Antunes F, Knobeloch KP, Owen MR, Naumann R, Decker T (2014). STAT1-cooperative DNA binding distinguishes type 1 from type 2 interferon signaling. Nat Immunol.

[CR6] Berenbaum F (2013). Osteoarthritis as an inflammatory disease (osteoarthritis is not osteoarthrosis!). Osteoarthr Cartil.

[CR7] Bogdanov R (1981). Bifurcations of a limit cycle for a family of vector fields on the plane. Sel Math Sov.

[CR8] Caron JP, Fernandes JC, Martel-Pelletier J, Tardif G, Mineau F, Geng C, Pelletier JP (1996). Chondroprotective effect of intraarticular injections of interleukin-1 receptor antagonist in experimental osteoarthritis. suppression of collagenase-1 expression. Arthritis Rheum.

[CR9] Chevalier X, Goupille P, Beaulieu AD, Burch FX, Bensen WG, Conrozier T, Loeuille D, Kivitz AJ, Silver D, Appleton BE (2009). Intraarticular injection of anakinra in osteoarthritis of the knee: a multicenter, randomized, double-blind, placebo-controlled study. Arthritis Care Res.

[CR10] Conaghan PG (2013). Osteoarthritis in 2012: parallel evolution of OA phenotypes and therapies. Nat Rev Rheumatol.

[CR11] Creamer P, Hochberg MC (1997). Osteoarthritis. Lancet.

[CR12] Domínguez-Hüttinger E, Ono M, Barahona M, Tanaka RJ (2013). Risk factor-dependent dynamics of atopic dermatitis: modelling multi-scale regulation of epithelium homeostasis. Interface Focus.

[CR13] Evans RC, Quinn TM (2006). Dynamic compression augments interstitial transport of a glucose-like solute in articular cartilage. Biophys J.

[CR14] Felson DT, Lawrence RC, Dieppe PA, Hirsch R, Helmick CG, Jordan JM, Kington RS, Lane NE, Nevitt MC, Zhang Y, Sowers M, McAlindon T, Spector TD, Poole AR, Yanovski SZ, Ateshian G, Sharma L, Buckwalter JA, Brandt KD, Fries JF (2000). Osteoarthritis: new insights. part 1: the disease and its risk factors. Ann Intern Med.

[CR15] Fernandes J, Tardif G, Martel-Pelletier J, Lascau-Coman V, Dupuis M, Moldovan F, Sheppard M, Krishnan BR, Pelletier JP (1999). In vivo transfer of interleukin-1 receptor antagonist gene in osteoarthritic rabbit knee joints: prevention of osteoarthritis progression. Am J Pathol.

[CR16] Fernandes JC, Martel-Pelletier J, Pelletier JP (2002). The role of cytokines in osteoarthritis pathophysiology. Biorheology.

[CR17] Goldring MB (2000). Osteoarthritis and cartilage: the role of cytokines. Curr Rheumatol Rep.

[CR18] Goldring MB (2000). The role of the chondrocyte in osteoarthritis. Arthritis Rheum.

[CR19] Graham JM, Ayati BP, Ding L, Ramakrishnan PS, Martin JA (2012). Reaction–diffusion-delay model for EPO/TNF-$$\alpha $$ interaction in articular cartilage lesion abatement. Biol Direct.

[CR20] Hawker G, Stewart L, French M, Cibere J, Jordan J, March L, Suarez-Almazor M, Gooberman-Hill R (2008). Understanding the pain experience in hip and knee osteoarthritis an OARSI/OMERACT initiative. Osteoarthr Cartil.

[CR21] Hedbom E, Huselmann HJ (2002). Molecular aspects of pathogenesis in osteoarthritis: the role of inflammation. Cell Mol Life Sci CMLS.

[CR22] Herald M (2010). General model of inflammation. Bull Math Biol.

[CR23] Hoff P, Buttgereit F, Burmester G, Jakstadt M, Gaber T, Andreas K, Matziolis G, Perka C, Rohner E (2013). Osteoarthritis synovial fluid activates pro-inflammatory cytokines in primary human chondrocytes. Int Orthop.

[CR24] Homandberg GA (1999). Potential regulation of cartilage metabolism in osteoarthritis by fibronectin fragments. Front Biosci.

[CR25] Homandberg GA, Hui F, Wen C, Purple C, Bewsey K, Koepp H, Huch K, Harris A (1997). Fibronectin-fragment-induced cartilage chondrolysis is associated with release of catabolic cytokines. Biochem J.

[CR26] Homandberg GA, Wen C, Hui F (1998). Cartilage damaging activities of fibronectin fragments derived from cartilage and synovial fluid. Osteoarthr Cartil.

[CR27] Izhikevich E (2006). Dynamical systems in neuroscience: the geometry of excitability and bursting.

[CR28] Jit M, Henderson B, Stevens M, Seymour RM (2005). TNF-$$\alpha $$ neutralization in cytokine-driven diseases: a mathematical model to account for therapeutic success in rheumatoid arthritis but therapeutic failure in systemic inflammatory response syndrome. Rheumatology.

[CR29] Kumar R, Clermont G, Vodovotz Y, Chow CC (2004). The dynamics of acute inflammation. J Theor Biol.

[CR30] Maly MR, Cott CA (2009). Being careful: a grounded theory of emergent chronic knee problems. Arthritis Care Res.

[CR31] Manicourt DH, Bevilacqua M, Righini V, Famaey JP, Devogelaer JP (2005). Comparative effect of nimesulide and ibuprofen on the urinary levels of collagen type II c-telopeptide degradation products and on the serum levels of hyaluronan and matrix metalloproteinases-3 and -13 in patients with flare-up of osteoarthritis. Drugs R&D.

[CR32] Martel-Pelletier J (2004). Pathophysiology of osteoarthritis. Osteoarthr Cartil/OARS Osteoarthr Res Soc.

[CR33] Martel-Pelletier J, Alaaeddine N, Pelletier JP (1999). Cytokines and their role in the pathophysiology of osteoarthritis. Front Biosci.

[CR34] Martel-Pelletier J, Boileau C, Pelletier JP, Roughley PJ (2008). Cartilage in normal and osteoarthritis conditions. Best Pract Res Clin Rheumatol.

[CR35] O’Hara BP, Urban JP, Maroudas A (1990). Influence of cyclic loading on the nutrition of articular cartilage. Ann Rheum Dis.

[CR36] Opal SM, DePalo VA (2000). Anti-inflammatory cytokines. Chest.

[CR37] Pearle AD, Warren RF, Rodeo SA (2005). Basic science of articular cartilage and osteoarthritis. Clin Sports Med.

[CR38] Poole AR (1993). Cartilage in health and disease. Arthritis Allied Cond Textb Rheumatol.

[CR39] Qvist P, Bay-Jensen AC, Christiansen C, Dam EB, Pastoureau P, Karsdal MA (2008). The disease modifying osteoarthritis drug (DMOAD): is it in the horizon?. Pharmacol Res.

[CR40] Roubille C, Pelletier JP, Martel-Pelletier J (2015) Drug/agent treatments for osteoarthritis: present and future. In: Kapoor M, Mahomed NN (eds) Osteoarthritis: pathogenesis, diagnosis, available treatments, drug safety, regenerative and precision medicine. Springer, Berlin, pp 191–210

[CR41] Sandell LJ, Aigner T (2001). Articular cartilage and changes in arthritis. an introduction: cell biology of osteoarthritis. Arthritis Res.

[CR42] Scanzello CR, Goldring SR (2012). The role of synovitis in osteoarthritis pathogenesis. Bone.

[CR43] Scanzello CR, Umoh E, Pessler F, Diaz-Torne C, Miles T, DiCarlo E, Potter HG, Mandl L, Marx R, Rodeo S, Goldring SR, Crow MK (2009). Local cytokine profiles in knee osteoarthritis: elevated synovial fluid interleukin-15 differentiates early from end-stage disease. Osteoarthr Cartil.

[CR44] Seymour RM, Henderson B (2001). Pro-inflammatoryanti-inflammatory cytokine dynamics mediated by cytokinereceptor dynamics in monocytes. Math Med Biol.

[CR45] Tetlow LC, Adlam DJ, Woolley DE (2001). Matrix metalloproteinase and proinflammatory cytokine production by chondrocytes of human osteoarthritic cartilage: associations with degenerative changes. Arthritis Rheum.

[CR46] Vincenti M, Brinckerhoff C (2002). Transcriptional regulation of collagenase (mmp-1, mmp-13) genes in arthritis: integration of complex signaling pathways for the recruitment of gene-specific. Arthritis Res.

[CR47] Wang X, Brouillette MJ, Ayati BP, Martin JA (2015). A validated model of the pro- and anti-inflammatory cytokine balancing act in articular cartilage lesion formation. Front Bioeng Biotechnol.

[CR48] Westacott CI, Sharif M (1996). Cytokines in osteoarthritis: mediators or markers of joint destruction?. Semin Arthritis Rheum.

[CR49] Wojdasiewicz P, Poniatowski LA, Szukiewicz D (2014) The role of inflammatory and anti-inflammatory cytokines in the pathogenesis of osteoarthritis. Mediators Inflamm 2014:561459. doi:10.1155/2014/561459 10.1155/2014/561459 PMC4021678 24876674

[CR50] Zhang L, Szeri A (2005). Transport of neutral solute in articular cartilage: effects of loading and particle size. Proc R Soc A Math Phys Eng Sci.

